# Fundamental Study of the Optical and Vibrational Properties of Fx-AZB@MOF systems as Functions of Dye Substitution and the Loading Amount

**DOI:** 10.1021/acs.langmuir.1c03482

**Published:** 2022-03-28

**Authors:** Markus Rödl, Alen Reka, Marko Panic, Alexander Fischereder, Marco Oberlechner, Thomas Mairegger, Holger Kopacka, Hubert Huppertz, Thomas S. Hofer, Heidi A. Schwartz

**Affiliations:** Institute of General, Inorganic and Theoretical Chemistry, University of Innsbruck, Innrain 80-82, A-6020 Innsbruck, Austria

## Abstract

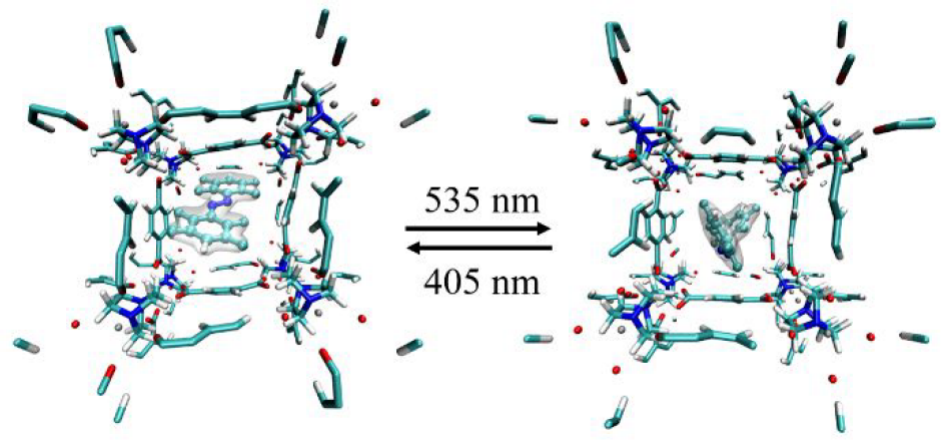

Controlling the switching efficiency of photoactive hybrid systems is an obligatory key prerequisite for systematically improving the design of functional materials. By modulating the degree of fluorination and the amount being embedded into porous hosts, the *E*/*Z* ratios of fluorinated azobenzenes were adjusted as both functions of substitution and the degree of loading. Octafluoroazobenzene (F8-AZB) and perfluoroazobenzene (F10-AZB) were inserted into porous DMOF-1. Especially for perfluoroazobenzene (F10-AZB), an immense stabilization of the *E* isomer was observed. In complementary molecular dynamics simulations performed at the DFTB (density functional tight binding) level, an in-depth characterization of the interactions of the different photoisomers and the host structure was carried out. On the basis of the resulting structural and energetic data, the experimentally observed increase in the amount of the *Z* conformer for F8-AZB can be explained, while the stabilization of *E*-F10-AZB can be directly related to a fundamentally different interaction motif compared to its tetra- and octafluorinated counterparts.

## Introduction

**Figure 1:**
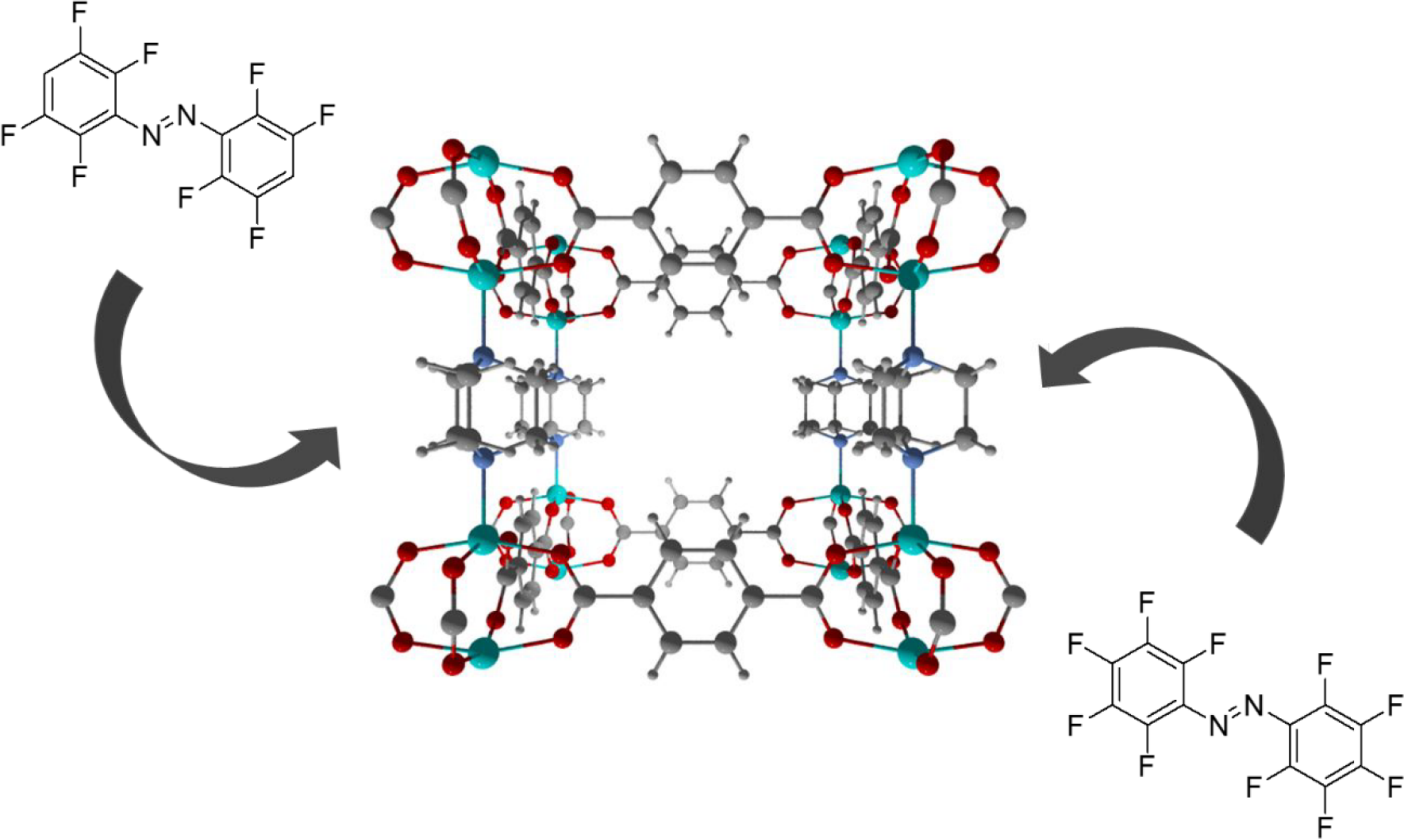
Structures of the *E* isomers of F8-AZB (left) and F10-AZB (right) as well as unloaded DMOF-1 (center).

In recent years, there has been rapid progress in the development of functional materials. In particular, hybrid materials are envisioned to be integrated into devices for data storage, energy conversion, and as sensor materials. In this respect, stimuli-responsive molecules have emerged as highly interesting candidates because their changes in conductivity, redox potential, magnetism, and optical properties represent on/off signals, which can be further exploited for the aforementioned applications. In the context of changing optical properties, photochromic molecules play an important role because they can be reversibly switched between their photoisomers by stimulus light.^1^ Unfortunately, these isomerization processes require a certain degree of steric freedom, which is why the photochromic response is mostly observed in solution rather than in pristine solids. To overcome this problem, insertion into a porous host matrix such as metal–organic frameworks (MOFs)^2^ simulates the dissolved state of the dye because single molecules are separated from each other. Hence, photoswitching is also enabled in the solid state. Metal–organic framework consist of metal cations or metal-oxo clusters, which are linked by an organic molecule with at least two functional groups to form networks with potential voids. Various photochromic dyes were incorporated into MOFs as noncovalently bound guests, including stilbenes,^3^ azobenzenes and functionalized derivatives,^4−10^ diarylethenes,^11,12^ spiropyrans,^13−16^ spirooxazines,^17^ fulgides,^18^ or donor–acceptor Stenhouse adducts (DASAs).^19^ For all resulting composites, photoswitching was enabled in the solid state, which makes these systems very interesting for further implementation as functional materials. Notably, the dye molecule can also be part of the linker backbone^20−23^ or can act as a linker pendant group.^24−32^ To understand the optical characteristics as a function of dye substitution, loading amount, and synthesis temperature, we recently started to investigate differently substituted spiropyrans^16^ as well as varying amounts of *ortho*-tetrafluoroazobenzene (F4-AZB)^9^ within the same host matrixes. Both studies showed a significant dependence of the resulting optical properties on the substitution pattern of the inserted guest molecule as well as on the amount being embedded. The latter work was motivated by a study presented by Kitagawa and co-workers:^4^ the authors embedded a nonsubstituted azobenzene into flexible MOF DMOF-1.^33^ UV light exposure resulted in the *E*-to-*Z* conversion (photostationary state of an *E*/*Z* ratio of 62:38) of the guest molecule and also in structural changes in the host material itself. Therefore, an even more significant impact was expected by the insertion of *ortho*-tetrafluoroazobenzene (F4-AZB). However, although F4-AZB exhibits almost quantitative switching (91% *Z* and 86% *E*) in acetonitrile,^34^ no such structural change of the host was observed when this dye was inserted into DMOF-1. Within the fundamental work presented in this study, we further deepen our study of azobenzenes with a varying degree of fluorination and different loading amounts inside DMOF-1. Octafluoroazobenzene (F8-AZB) and perfluoroazobenzene (F10-AZB) were inserted into DMOF-1 in varying amounts, and the optical characteristics of the resulting composite materials were analyzed both as a function of fluorination and as a function of the degree of loading to account for possible guest–guest interactions. Figure 1 depicts the structures of applied guest molecules F8-AZB and F10-AZB and host structure DMOF-1.

Both F8-AZB and F10-AZB show switching yields of up to 92% inside acetonitrile for the *E* and *Z* isomers, which is even higher than the amounts observed for F4-AZB.^34^ For this reason, the degree of fluorination is expected to influence the switchability of these azobenzenes within DMOF-1 in the sense that photoswitching becomes even more efficient than for azobenzene and *ortho*-tetrafluoroazobenzene. Furthermore, light-induced changes in the diffraction patterns should then be visible, as already shown by Kitagawa and co-workers.

To correlate the experimental results with the interaction between the guest molecules and the host structure, several theoretical investigations have been carried out. Similar to the previous study focused on F4-AZB@DMOF-1,^9^ a molecular dynamics (MD) simulation approach describing the system at an elevated temperature was preferred over the calculation of simple minimum structures corresponding to 0 K treatment. Previously, it was shown that the interaction motif of *Z*-F4-AZB involved only BDC^2–^ (i.e., terephthalate anion) units associated with a single unit cell of the host structure, forming a square coordination cavity. On the other hand, the *E*-F4-AZB conformer preferably interacts with BDC^2–^ residues associated with two neighboring unit cells of the host structure. To properly describe the guest–host interactions in the latter case, a 2 × 2 × 2 supercell corresponding to [Zn_2_(BDC)_2_(DABCO)]_8_ (dabco = 1,4-diazabicyclo[2.2.2]octane) proved to be the minimum size to represent the host structure, which also reduces the effects of guest–guest interactions arising from the periodic treatment of the system. However, this system size (total number of electrons of 2320 just in case of the MOF) proved too demanding to execute an MD simulation of about 100 ps at the density functional theory (DFT) level. Recently, density functional tight binding (DFTB) approaches^35,36^ were found to provide a highly successful alternative to DFT calculations. Despite providing a simplified, semiempirical description of the interactions, a DFTB MD simulation approach delivered an accurate description of pristine DMOF-1 when compared to experimental reference data as well as a detailed structural description of *E*/*Z*-F4-AZB@DMOF-1 that correlated well with the experimental findings.^9^ For this reason, this study employs the same simulation approach to investigate the binding properties of *E*/*Z*-F8-AZB and *E*/*Z*-F10-AZB embedded in the DMOF-1. In addition to MD simulations of the guest@host systems, the comparison of theoretically and experimentally determined difference infrared (IR) spectra proved to be a valuable tool to correlate the vibrational properties of the individual conformers associated with the photoswitching mechanism.^18^ This strategy was therefore also applied in this study to trace the switching processes of the inserted dye molecules.

## Experimental Section

Commercially available 1,4-diazabicyclo[2.2.2]octane (Sigma-Aldrich), 2,3,5,6-tetrafluoroaniline (Apollo Scientific), pentafluoroaniline (abcr), *N*,*N′*-dimethylformamide (n.i.), chloroform (ZEUS), lead(IV) acetate (Alfa Aesar), terephthalic acid (Alpha Aesar), and [Zn(NO_3_)_2_] (n.s.) were used without any further purification.

### Octafluoroazobenzene Synthesis

2,3,5,6-Tetrafluoroaniline (451.0 mg, 2.73 mmol) was dissolved in chloroform (25 mL), and lead(IV) acetate (3.03 g, 6.83 mmol) was added. The color of the suspension changed from colorless to orange. The suspension was stirred overnight at room temperature. The crude residue was filtered, and the solution was washed three times with glacial acetic acid (50%). The organic phase was dried over sodium sulfate. The solvent was removed under reduced pressure. The residue was purified using column chromatography (cyclohexane) and obtained as an orange-red salt. The purity of octafluoroazobenzene was confirmed by ^1^H and ^19^F NMR spectroscopy (Figures S1 and S2, Supporting Information).

^1^H NMR (400 MHz, CDCl_3_): δ/ppm = 7.28–7.20 (m, 2H, *E-4H*, *4′H*), 7.11–7.03 (m, 2H, *Z-4H*, *4′H*). ^19^F NMR (400 MHz, CDCl_3_): δ/ppm = −136.1 (m, 4F, *Z-3F*, *3′F*, *5F*, *5′F*), −138.0 (m, 4F, *E-3F*, *3′F*, *5F*, *5′F*), −147.3 (m, 4F, *Z-2F*, *2′F*, *6F*, *6′F*), −149.6 (m, 4F, *E-2F*, *2′F*, *6F*, *6′F*).

### Perfluoroazobenzene Synthesis

Pentafluoro aniline (500.0 mg, 2.73 mmol) was dissolved in toluene (30 mL), and lead(IV) acetate (3.03 g, 6.83 mmol) was added. The color of the suspension changed from colorless to orange. The suspension was stirred overnight at room temperature. The crude residue was filtered, and the solution was washed three times with glacial acetic acid (50%). The organic phase was dried over sodium sulfate. The solvent was removed under reduced pressure. The residue was purified using column chromatography (dichlormethane/cyclohexane 3:7) and obtained as an orange salt. The purity of perfluoroazobenzene was confirmed by ^19^F NMR spectroscopy (Figure S3, Supporting Information).

^19^F-NMR (400 MHz, CDCl_3_): δ/ppm = −161.2 (m, 4F, *E-2F*, *2′F*, *6F*, *6′F*), −158.0 (m, 4F, *Z-2F*, *2′F*, *6F*, *6′F*), −150.3 (m, 2F, *Z-4F*, *4′F*), −148.2 (m, 6F, *Z-3F*, *3′F*, *4F*, *4′F, 5F*, *5′F*), −146.2 (m, 4F, *Z-3F*, *3′F*, *5F*, *5′F*)

### DMOF-1 Synthesis

Zn(NO_3_)_2_·6 H_2_O (125.0 mg, 0.42 mmol), terephthalic acid (70.0 mg, 0.42 mmol), and dabco (1,4-diazabicyclo[2.2.2]octane; 20.0 mg, 21.0 mmol) were mixed with DMF (dimethylformamide) (3–5 mL) in an 8 mL Teflon-lined autoclave. The mixture was heated (120 °C, 2 days) in an oven and then cooled to room temperature. The resulting colorless powder was filtered, washed with a small amount of DMF, and dried in air overnight. To remove embedded DMF molecules, the residue was heated under reduced pressure (120 °C, 24 h) and stored under an argon atmosphere. The phase purity of DMOF-1 was checked with XRPD (Figure S4, Supporting Information).

### Preparation of oF_*x*_-AZB@DMOF-1 Systems

F8-AZB/DMOF-1 molar ratios of 3:1, 1:1, and 0.125:1 were mixed under an argon atmosphere. The resulting homogeneous powder was placed in a small glass vessel inside a Schlenk tube and heated to 55 °C under a reduced pressure of ∼9.4 × 10^–2^ mbar for several hours in the dark. The excess F8-AZB resublimed at the top of the glass tube. To prevent the absorption of water and decomposition upon contact with air and moisture, all compounds were stored in a glovebox under an argon atmosphere. Furthermore, the sample was kept in the dark to avoid any undesired switching processes.

### Preparation of pF_*x*_-AZB@DMOF-1 Systems

F10-AZB/DMOF-1 molar ratios of 3:1, 1:1, and 0.125:1 were mixed under an argon atmosphere. The resulting homogeneous powder was placed in a small glass vessel inside a Schlenk tube and heated to 65 °C under reduced pressure of ∼9.4 × 10^–2^ mbar for several hours in the dark. The excess F8-AZB resublimed at the top of the glass tube. To prevent the absorption of water and decomposition upon contact with air and moisture, all compounds were stored in a glovebox under an argon atmosphere. Furthermore, the sample was kept in the dark to avoid any undesired switching processes.

The phase purity of the resulting hybrid materials was checked by XRPD measurements. The XRPD patterns of the dilutions are shown in Figures S5 and S6, Supporting Information.

### X-ray Powder Diffraction

To check the purity of the crystalline samples, laboratory measurements were carried out on a Stoe Stadi P diffractometer (Stoe, Darmstadt, Germany) in transmission geometry with Mo Kα1 radiation (λ = 0.7093 Å) by utilizing a focusing Ge(111) primary beam monochromator and a Mythen 2 DCS4 detector. The measurement was performed in the 2θ range of 2.0–40.4° with a step size of 0.015°. The respective powder was sealed in a glass capillary under an argon atmosphere to prevent the absorption of humidity. To follow light-induced structural guest-to-host transmission, XRPD patterns were collected after 15 min of 405 and 535 nm irradiation. For this purpose, the capillary was rotated further on the goniometer head so that the light exposure was as even as possible. Subsequently, the diffraction pattern was collected. All of these steps were performed in the dark to prevent any daylight from reaching the sample. For the illumination of the hybrid materials, a Prizmatix PRI FC5-LED-WL (output from five high-power fiberglass-coupled LEDs with a potentiometer for manual power control) was used.

### Reflection Spectroscopy

Reflection spectra of the Fx-AZB@DMOF-1 systems were recorded using an Agilent Cary 5000 UV–vis–NIR spectrophotometer. Therefore, the powder was placed in a sample holder under an argon atmosphere to prevent the absorption of humidity. Spectra were recorded in the range of 200 to 700 nm before and after irradiation (λ = 405 or 535 nm, 5 min). Detailed information on the irradiation processes is given for the respective spectra. For the illumination of the hybrid materials, a Prizmatix PRI FC5-LED-WL (output from five high-power fiberglass-coupled LEDs with a potentiometer for manual power control) was used.

### Liquid-State NMR Spectroscopy

^1^H NMR spectra were collected on a 300 MHz Bruker Avance DPX NMR spectrometer equipped with a 5 mm broadband probe. The solvent served as an internal reference (δ_H_(CDCl_3_) = 7.24 ppm, δ_H_(DMSO*-d*_*6*_) = 2.50 ppm). ^19^F spectra were recorded on a 400 MHz Bruker Avance 4 Neo spectrometer. All measurements were carried out at room temperature and processed with *MestReNova 9.0.1-13254*. For liquid-state NMR measurements on F8-AZB and F10-AZB, small amounts of *xF*-AZB were dissolved in CDCl_3_. For liquid-state NMR measurements on the hybrid systems, approximately 3.0 mg of F8-AZB_*x*_@DMOF-1 was digested in 0.5 mL of DMSO-*d*_*6*_ and 25 μL of DCl was added. Directly after the addition of DCl, the sample was placed in an NMR glass tube. The spectra were recorded ca. 2 min after treatment with DCl. To determine the composition, characteristic proton signals were integrated and related to each other.

### Infrared Spectroscopy

To understand the occurring host–guest and guest–guest interactions, all compounds and pure DMOF-1 were analyzed via IR spectroscopy. The measurements were carried out on a Bruker Alpha II FT-IR spectrometer under an argon atmosphere to prevent the absorption of humidity. The hybrid materials were prepared as follows: one-half of a spatula tip of the sample was thoroughly ground with two spatulas of KBr. Afterward, the mixture was pressed for 30 min in a pressure apparatus with a set pressure of ∼2 tons, yielding a thin, transparent light-colored pellet. The pellet was then placed in the IR spectrometer sample holder. Scans were performed in the range of 360–4000 cm^–1^ with a resolution of 2 cm^–1^ and 90 scans per sample. The background was determined by preparing a pure KBr pellet and measuring it with the same instrument settings as used for the sample. Furthermore, IR spectra of the irradiated samples (violet and green light exposure) were collected. For this purpose, the samples were irradiated for 5 min. All measurements were carried out at room temperature and evaluated with the OPUS version 8.2 build 8, 2, 28(20190310) program, copyright Bruker Optic GmbH. For the illumination of the hybrid materials, a Prizmatix PRI FC5-LED-WL (output from five high-power fiberglass-coupled LEDs with a potentiometer for manual power control) was used. The spectra can be found inFigures 6 and 7 as well as in Figures S7–S12 in the Supporting Information, where they are compared to nonloaded DMOF-1. To quantify the *E*/*Z* ratios, the heights of characteristic *E*/*Z* peaks were related to each other before and after irradiation with violet and green light, respectively.

**Figure 2:**
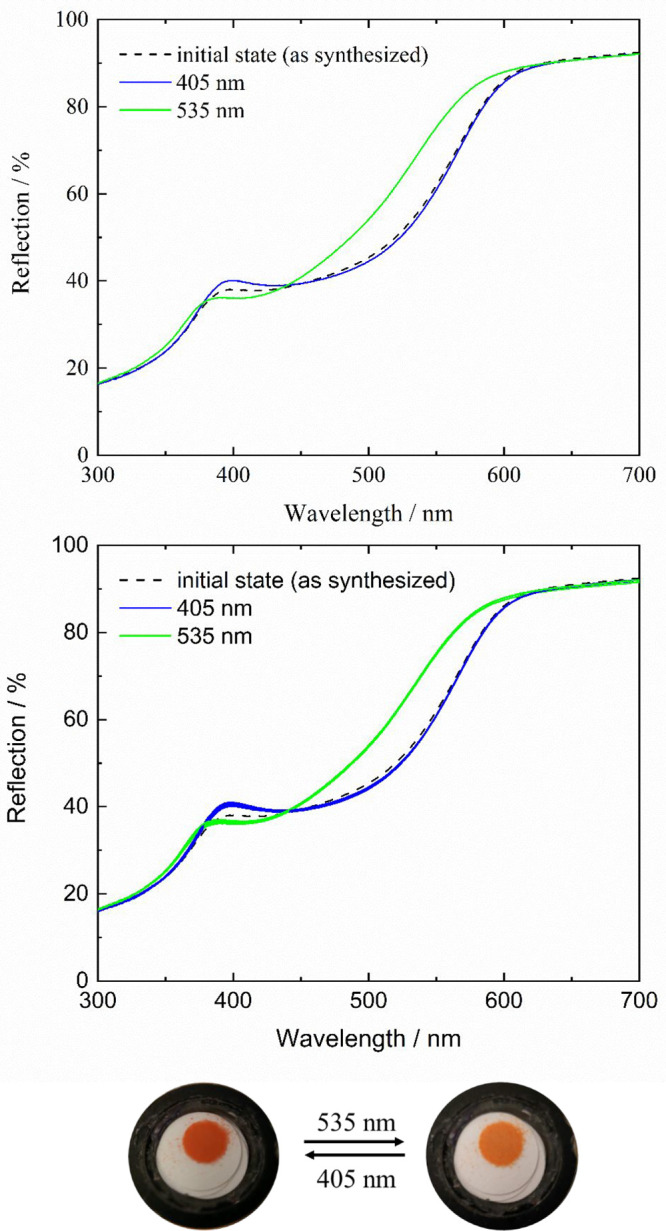
(Top) UV/vis reflectance spectra of F8-AZB_0.125_@DMOF-1 before (dashed black line) and after irradiation with violet (blue line) and green light (green line). (Center) UV/vis reflectance spectra of F8-AZB_0.125_@DMOF-1 over 10 switching cycles. (Bottom) Light-induced color change of F8-AZB@DMOF-1 upon irradiation with violet (λ = 405 nm) and green light (λ = 535 nm).

**Figure 3:**
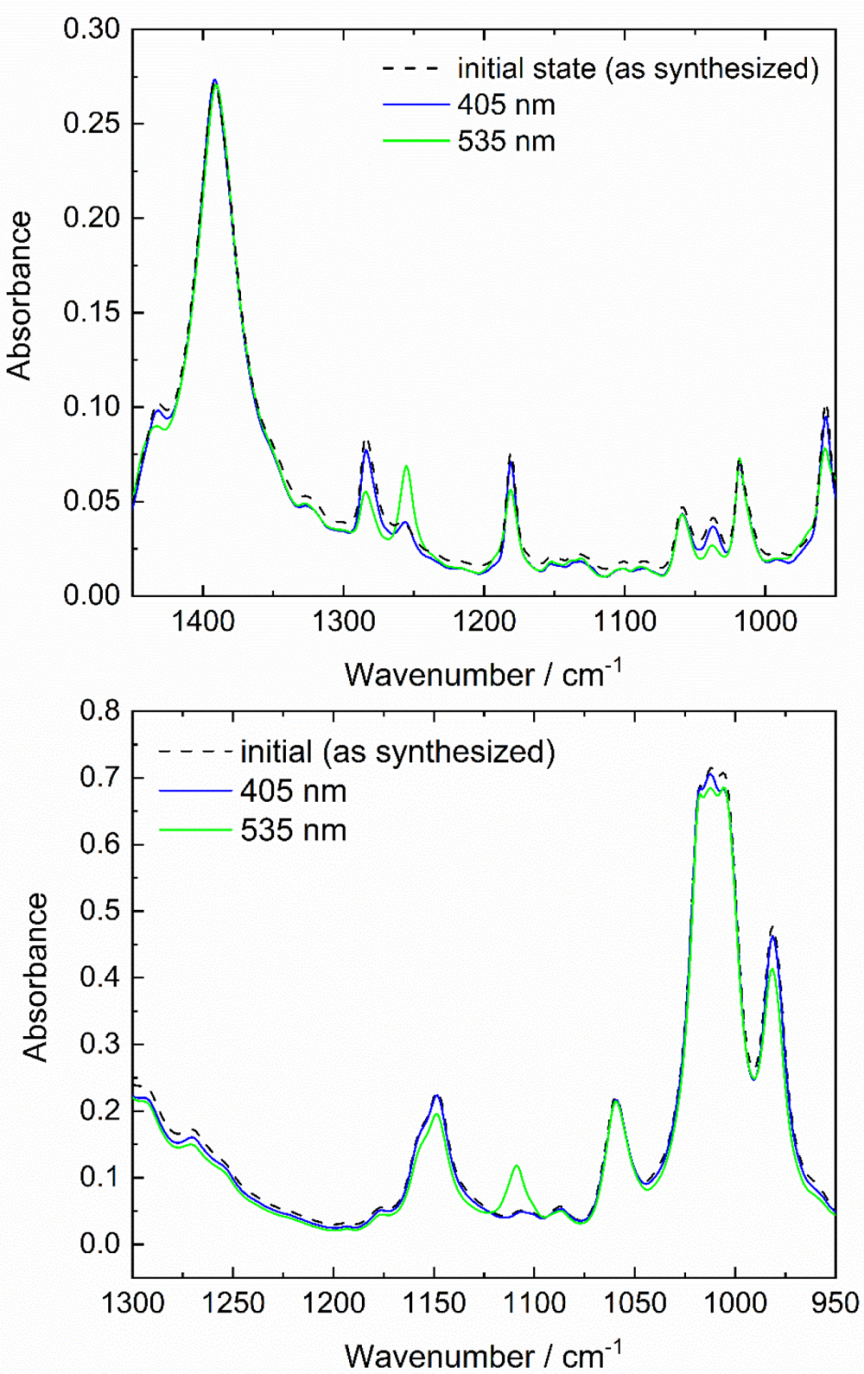
IR spectra of F8-AZB@DMOF-1 (top) and F-10-AZB@DMOF-1 (bottom) before (dashed black line) and after irradiation with violet (blue line) and green light (green line).

**Figure 4:**
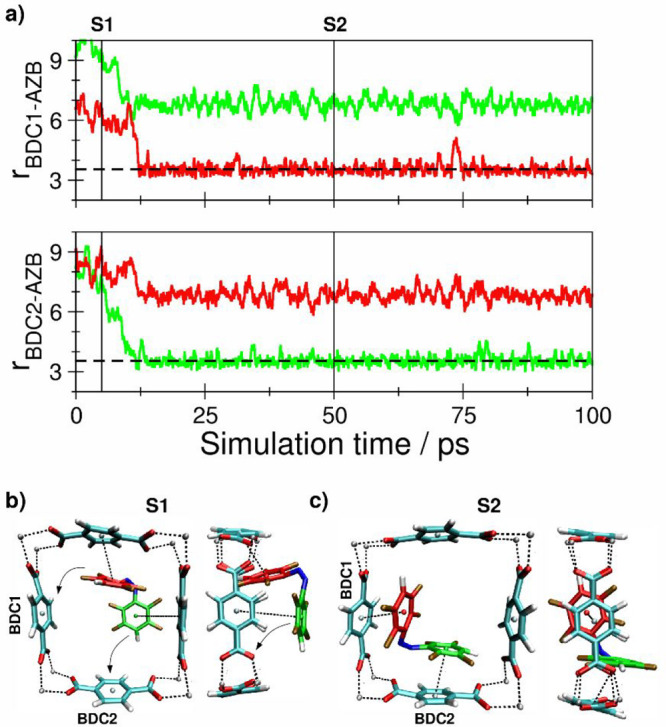
(a) Key distances between *Z*-F8-AZB and BDC^2–^ groups of the DMOF-1 host lattice based on the centroids of the aromatic units determined as the average over the respective carbon atoms. (b and c) Snapshots displaying representative configurations of the *Z*-F8-AZB@DMOF-1 interaction taken from the simulation trajectory. The associated time steps are marked as S1 and S2 in the time series.

**Figure 5:**
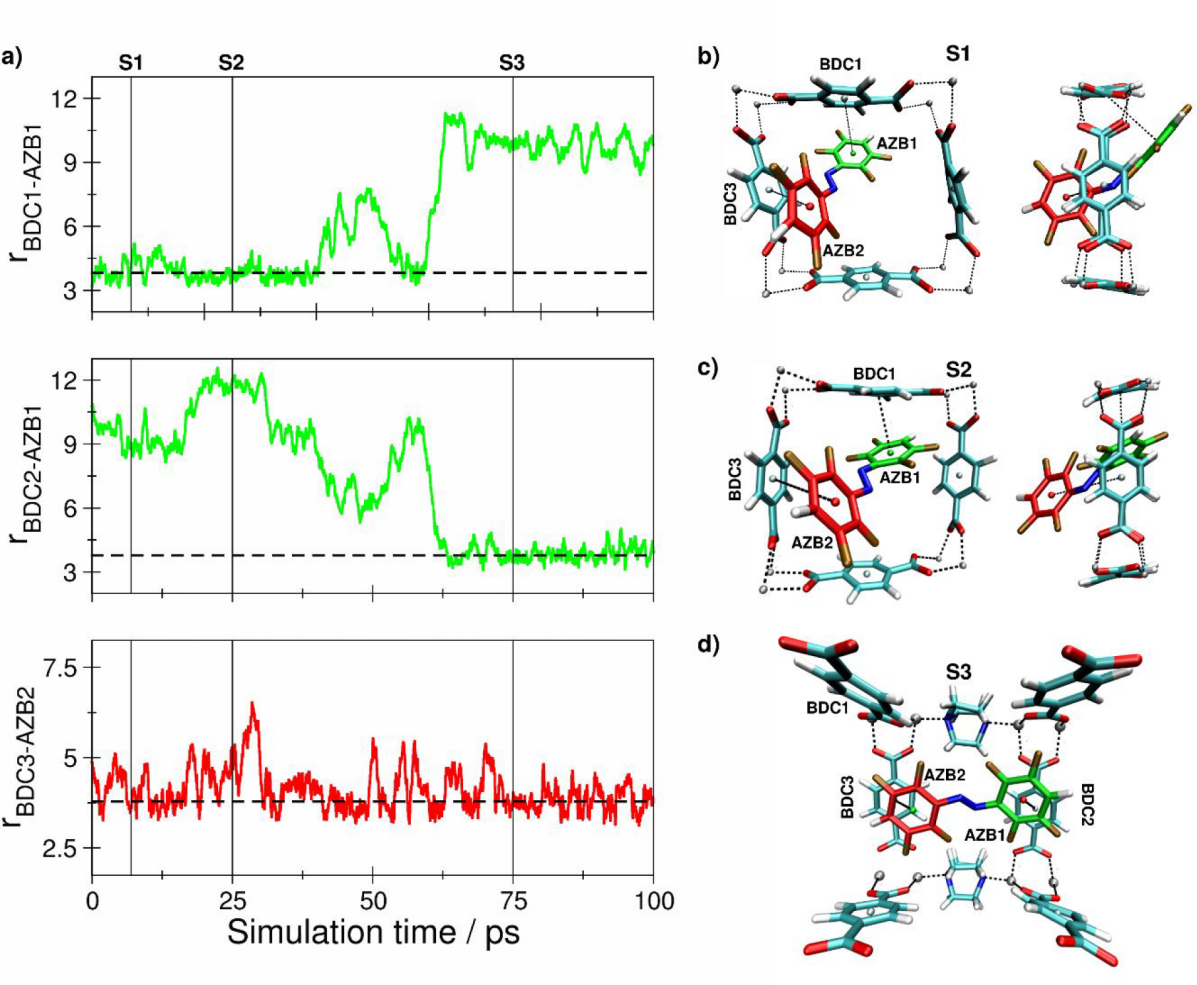
(a) Key distances between *E*-F8-AZB and BDC^2–^ groups of the DMOF-1 host structure based on the centroids of the aromatic units determined as the average over the respective carbon atoms. (b–d) Snapshots displaying representative configurations of the *E*-F8-AZB@DMOF-1 interaction taken from the simulation trajectory. The associated time steps are marked as S1 to S3 in the time series.

**Figure 6:**
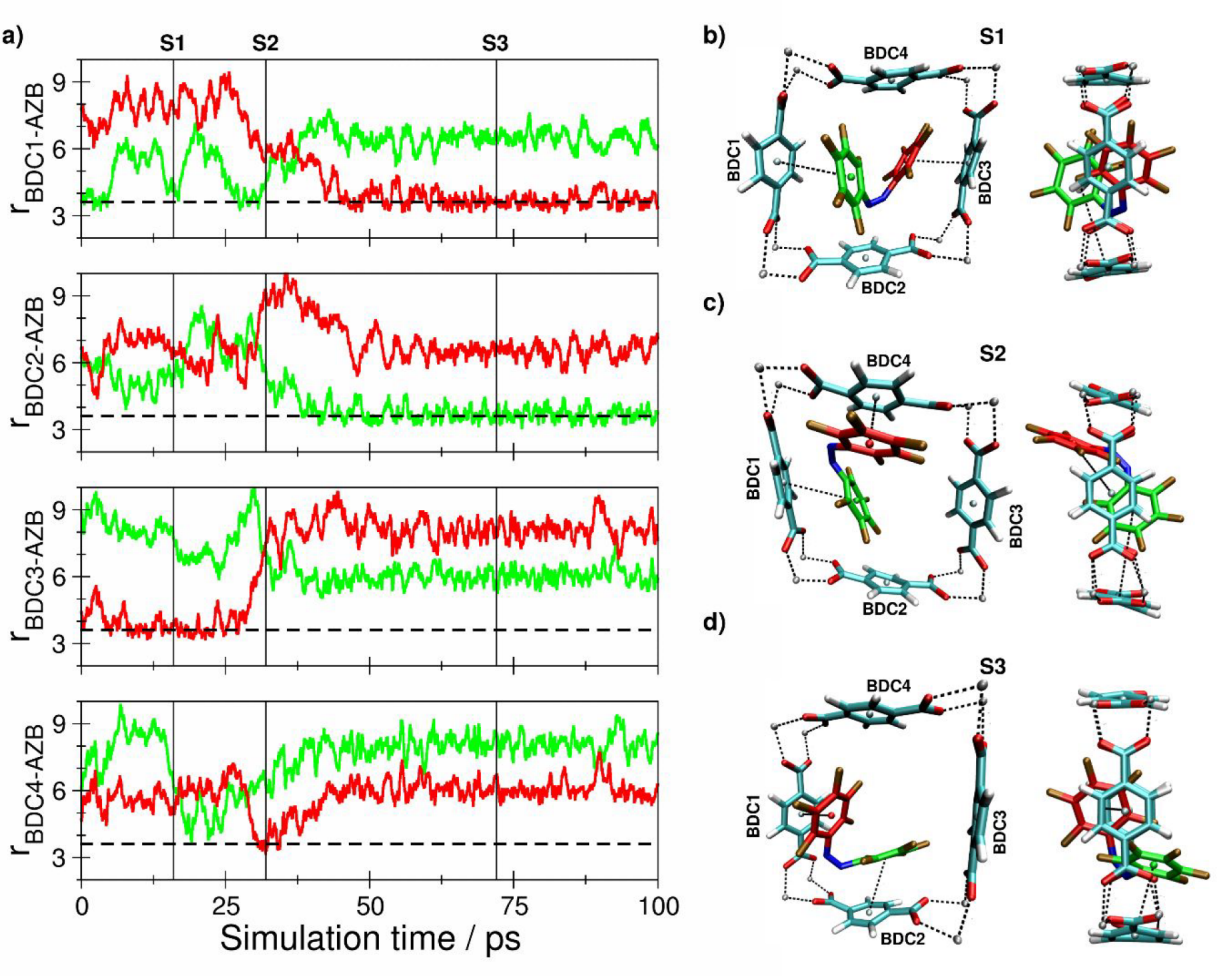
(a) Key distances between *Z*-F10-AZB and BDC^2–^ groups of the DMOF-1 host structure based on the centroids of the aromatic units determined as the average over the respective carbon atoms. (b–d) Snapshots displaying representative configurations of the *Z*-F10-AZB@DMOF-1 interaction taken from the simulation trajectory. The associated time steps are marked as S1 to S3 in the time series.

**Figure 7:**
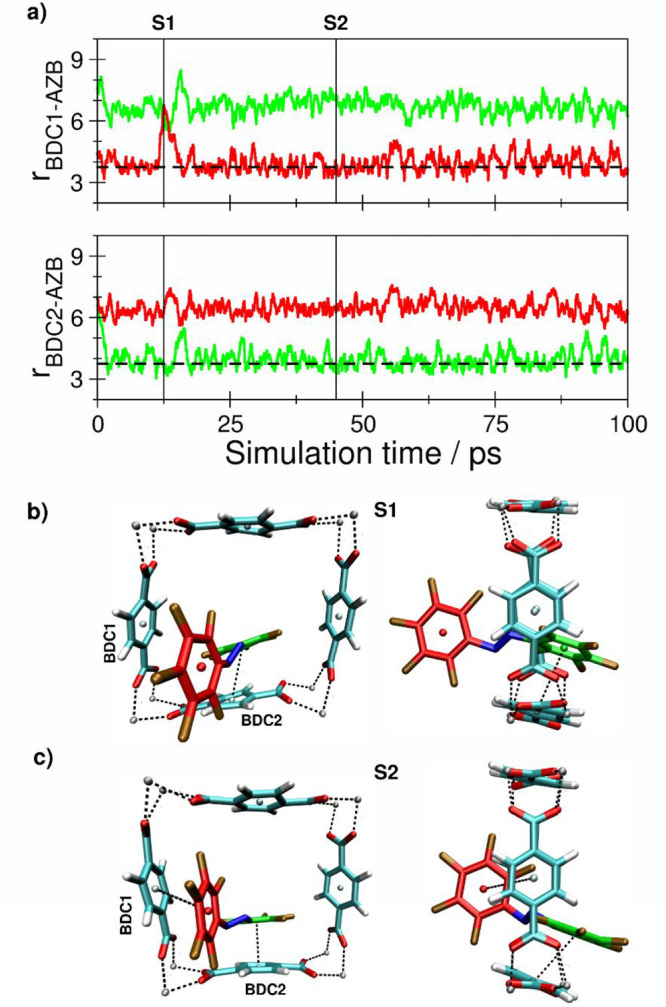
(a) Key distances between *E*-F10-AZB and BDC^2–^ groups of the DMOF-1 host structure based on the centroids of the aromatic units determined as averages over the respective carbon atoms. (b and c) Snapshots displaying representative configurations of the *E*-F10-AZB@DMOF-1 interaction taken from the simulation trajectory. The associated time steps are marked as S1 and S2 in the time series.

### DFTB MD Simulation Protocol

The density functional tight binding (DFTB) approach^35,36^ is based on a Taylor series of the Kohn–Sham energy known from density functional theory (DFT) with respect to the equilibrium density, enabling a parametrization of simple tight binding (TB) Hamiltonians^39^ with respect to high-level DFT reference data. As in the previous study of *E*/*Z*-F4-AZB,^9^ the self-consistent charge density functional tight binding (SCC DFTB)^40,41^ method in conjunction with the 3ob parameter set^42−44^ as implemented in the DFTB+ package^45^ was employed to describe the intermolecular forces under periodic boundary conditions, thereby considering each atom as irreducible. Monkhorst–Pack sampling on a (2 × 2 × 2) grid was employed for the integration along the axes of the Brillouin zone. As defined in the parametrization of the 3ob parameter set, damping factor χ_XH_ was applied to damp all interactions between hydrogen and non-hydrogen atoms.^44^ In addition, the Grimme D3 correction^46^ was employed to improve the description of dispersion interactions in the system. To achieve a conversion in the energy of ≤10^–6^ Hartree, the convergence criterion of the SCC error was set to 10^–4^. Earlier, the DFTB+ package has been interfaced to our in-house QM/MM MD simulation program^47−49^ to execute the individual steps of the molecular dynamics simulation. To integrate the equations of motion, the velocity-Verlet algorithm^50,51^ was employed in conjunction with the Shake/Rattle algorithms^52,53^ to uphold holonomic bond constraints applied to all hydrogen-containing bonds, thus enabling an MD time step of 2.0 fs. All simulations were executed in the isothermal–isobaric (NPT) ensemble employing the Nose-Hoover chain thermostat^54^ with a chain length of 5 in conjunction with a Berendsen manostat^55^ using a relaxation time of 10 ps. To take the tetragonal character of the unit cell into account, semi-isotropic pressurization was applied; i.e., the coupling along the *a* and *b* directions was realized independently from the adjustments along the *c* axis. The visualization of the simulation trajectories and the generation of screenshots have been performed using the VMD package.^56^

All simulations containing a single *E*- or *Z*-F8- or F10-AZB molecule have been started from the same equilibrated DMOF-1 structure as used in the earlier study focused on *E*/*Z*-F4-AZB.^9^ The respective molecule was inserted at a random position upholding a minimum distance of 1.25 Å from all atoms associated with the MOF structure. To confirm the unexpected binding motif observed in the case of *E*-F10-AZB, a fifth DFTB MD simulation has been carried out, thereby employing the final configuration obtained in the simulation of *E*-F8-AZB@DMOF-1 as a structural template for the starting configuration. After the insertion of the respective guest molecule, each system has been equilibrated for 12.5 ps (6250 MD steps) followed by a sampling period of 100 ps (50 000 MD steps).

To assess the interaction energy *U*_int_ between the host and the guest molecule, simulations of isolated *E*/*Z*-F8- and *E*/*Z*-F10-AZB were executed, employing the same simulation setup (albeit without a pressure coupling). Again, simulation times of 12.5 and 100 ps were employed for the equilibration and sampling phases. The resulting time series of the calculated total potential of the guest molecule has been averaged, yielding ⟨*U*_guest_⟩. The instantaneous guest–host interaction energy *U*_int_ is then evaluated as follows
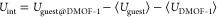
1with *U*_guest@DMOF-1_ being the instantaneous potential obtained from the respective DFTB MD calculation at a particular time step and ⟨*U*_DMOF-1_⟩ being the averaged potential obtained via a separate MD simulation of the pristine MOF under identical conditions. To estimate the associated average interaction energy ⟨*U*_int_⟩, averaging over the last 25 ps of the simulation trajectory has been performed to ensure the formation of a stable binding motif between the host and the investigated guest molecule.

### Harmonic Frequency Calculations

In addition to the DFTB MD simulations, vibrational difference spectra of the guest molecules have been calculated at the DFT level by employing the B3LYP exchange-correlation functional^57^ in conjunction with the 6-31G(d,p) basis set^58−60^ as implemented in the *Gaussian 16* quantum chemical calculation software.^61^ To improve the description of dispersion interactions, the Becke-Johnsen D3 correction was applied.^62^ To approximate the influence of the surrounding material, the SMD method (solvation model density) was employed using a permittivity of 28.735 corresponding to ethanol as successfully applied in a previous study.^18^ The calculated vibrational frequencies ν_calc_ have been scaled by the procedure reported by Katari and co-workers,^63^ which, instead of a constant scaling factor, employs a linear equation of the form
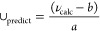
2Parameters *a* and *b* were set to 1.0726 and −63.0 cm^–1^, respectively, as recommended for the B3LYP/6-31G(d,p) level of theory. The respective equilibrium geometries and the unscaled vibrational frequencies ν_calc_ are listed in the Supporting Information, Tables S1–S8.

## Results

### Successful Incorporation of Fx-AZB inside DMOF-1

The synthesis of the Fx-AZB@MOF systems was performed via a gas-phase loading process, which has proven to be the most efficient strategy to exclusively study host–guest and guest–guest interactions without the potential presence of any solvent molecule. Three dilutions were chosen (molar ratios of guest/host of 0.125:1, 1:1, and 3:1) to account for both host–guest (small numbers of guest molecules being inserted) and guest–guest interactions (maximum guest loading inside the MOF pores). The resulting composite materials were analyzed following the ICE-principle protocol,^17^ where ICE stands for incorporation, composition, and effects. The successful formation of the Fx-AZB@DMOF-1 systems was confirmed via X-ray powder diffraction (XRPD) measurements. The respective XRPD patterns can be found in Figures S5 and S6 in the Supporting Information. Here, a superposition of the orthorhombic and tetragonal forms of DMOF-1 is observed, which is consistent with the data obtained previously using F4-AZB as a guest molecule.^9^ Because no additional reflections of F8-AZB and F10-AZB are present, nonembedded crystalline F8-AZB and F10-AZB can be excluded. However, nonembedded amorphous F8-AZB and F10-AZB cannot be traced via XRPD. In a previous study on a nitrosubstituted spiropyran,^14^ X-ray photoelectron spectroscopy (XPS) proved to be a powerful tool for distinguishing between noninserted and inserted guest molecules. Here, a significant broadening of the nitrogen signal was found for the embedded species, whereas this signal is sharp for surface-adsorbed spiropyran. XPS measurements, however, are performed in ultrahigh vacuum (UHV), which is a drawback for azobenzenes: even sublimation out of the pores was observed under these measurement conditions. In ongoing work, solid-state NMR and total scattering coupled to the PDF (pair distribution function) will be utilized to overcome the limitations of common diffraction methods.

### Determination of the Compositions of F8-AZB_*x*_@DMOF-1 and F10-AZB_*x*_@DMOF-1 Systems

The composition of the obtained F8-AZB_*x*_@DMOF-1 systems was performed via liquid-state NMR spectroscopy following the procedure previously described for diarylethenes,^11,12^ spirooxazines,^17^ spiropyrans,^16^ F4-AZB,^9^ and fulgides^18^ inside different MOF hosts. For this, the respective compound was digested in DMSO-*d*_*6*_ and DCl. Subsequently, the peak area of the terephthalic acid protons was related to the signals of the present *E*- and *Z*-isomer protons of F8-AZB. In Figures S13 to S15 in the Supporting Information, the corresponding ^1^H NMR spectra are depicted. Notably, the proton signals for the *Z* isomer overlap with the signal of the MOF linker molecule. For the calculations of the composition, the *E*/*Z* ratios in the initial state for the F8-AZB@DMOF-1 systems were considered (Table 2). Detailed information in the calculation process is given in the Supporting Information (Table S9, Supporting Information). Table 1 lists the determined compositions for the F8-AZB_*x*_@DMOF-1 systems. Because F10-AZB lacks protons in its structure, liquid-state NMR spectroscopy is not a suitable method for determining the composition of the obtained composite materials. Therefore, IR spectroscopy was applied for these systems. The calculated compositions are given in Table 1. Again, detailed information on the calculation process is given in Table S10 in the Supporting Information.

**Table 1:** Compositions of F8-AZB_*x*_@DMOF-1 and F10-AZB_*x*_@DMOF-1 Systems Taking into Account the Isomer Ratios (Table 2) in the Initial State

	calculated value	calculated value
quantity used	F8-AZB_*x*_@DMOF-1	F10-AZB_*x*_@DMOF-1
*x* = 0.125	**x** = 0.125	*x* = 0.12
**x** = 1	*x* = 1	*x* = 1.77
*x* = 3	*x* = 1.24	*x* = 1.19

As visible in Table 1, all calculated values are reasonable. The only exception is found for the F10-AZB@DMOF-1 system with *x* = 1 as the quantity used. Here, the calculated dye-to-MOF ratio is almost twice as high as expected, although the exact molar ratios were weighed in and measurements were performed several times. An inhomogeneous loading is assumed, which will be further investigated in ongoing studies.

### Probing the Optical Characteristics as a Function of Fluorination and the Degree of Loading

Upon visible light irradiation, both F8-AZB and F10-AZB can be converted between their *E* and *Z* isomers in solution, e.g., when dissolved in acetonitrile.^34^ Here, *E*-F8-AZB shows an absorption maximum at 303 nm for the π–π* transition and at 456 nm for the n−π* transition, whereas these maxima shift to lower wavelengths for the *Z* isomer, which are located at 240 nm for the π–π* transition and at 413 nm for the n−π* transition. For *E*-F10-AZB, the π–π* transition occurs at 310 nm and the n−π* transition occurs at 453 nm. Upon violet light irradiation, the *Z* isomer is generated, which shows an absorption maximum at 413 nm for the n−π* transition. To probe the photochromic response of F8-AZB and F10-AZB inside DMOF-1, UV/vis reflection spectra were recorded before and after irradiation with violet (λ = 405 nm) and green light (λ = 535 nm), respectively. Furthermore, the reversibility of switching was studied by repetitive visible light exposure. For the irradiation process, 5 min of irradiation was found to be sufficient because no change after 4 min of light exposure was found (Figure S16 top and bottom, Supporting Information). In Figure 2, top, the reflection spectra of F8-AZB_0.125_@DMOF-1 before and after irradiation and over 10 switching cycles are shown. Switching cycles of F8-AZB@DMOF-1 and F8-AZB_3_@DMOF-1 are depicted in Figure S17 in the Supporting Information. These data clearly show the reversibility of switching. In the following section, the optical characteristics of F8-AZB_0.125_@DMOF-1 will be described as an example for all F8-AZB_*x*_@DMOF-1 systems.

In the initial state, a reflection minimum at ∼423 nm is found, which corresponds to a mixture of the *E* isomer and Z isomer (both n−π* transitions) (Figure 2 top, dashed black line). Upon violet light irradiation, this reflection minimum shifts to approximately 445 nm (Figure 2 top, blue line), whereas green light exposure causes a shift of this minimum to approximately 410 nm (Figure 2 top, green line). This minimum is the result of the *Z*-isomer population. Therefore, the photochromic response of F8-AZB is retained when embedded in DMOF-1. Upon repetitive violet and green light exposure, the resulting reflection spectra are identical, which is shown in Figure 2, bottom. The *E*-to-*Z* conversion is reversible over 10 switching cycles without any fatigue, as visible in the overlapping spectra for the *E* and *Z* isomers. The significant changes in the reflection characteristics upon UV light irradiation are also visible by the naked eye. Figure 2, bottom depicts the light-induced color changes of the F8-AZB@DMOF-1 sample.

Analogous to F8-AZB, the optical characteristics of F10-AZB inside DMOF-1 were elucidated. The resulting reflection spectra of F10-AZB_0.125_@DMOF-1, F10-AZB@DMOF-1, and F10-AZB_3_@DMOF-1 are shown in Figure S18 in the Supporting Information. For F10-AZB_0.125_@DMOF-1 in the nonirradiated state, a reflection minimum at ∼445 nm is found (Figure S19 top, dashed black line). This minimum corresponds to the n−π* transition of the *E* isomer and remains unchanged upon violet light exposure (Figure S18, top, blue line). Upon subsequent green light irradiation, a new reflection minimum at ∼416 nm appears and the n−π* transition of the *E* isomer vanishes (Figure S18, top, green line). The new minimum originates from the n−π* transition of the *Z* isomer of F10-AZB. The generation of both the *E* and *Z* conformers is reversible over 10 switching cycles, which is again visible in the overlapping spectra shown in Figure S18, bottom. These optical characteristics are also observed for F10-AZB@DMOF-1 and F10-AZB_3_@DMOF-1 (Figure S19, Supporting Information). Conclusively, embedment into the MOF host enables reversible photoswitching for both F8-AZB and F10-AZB, independently of the amounts being embedded. To quantify the *E* and *Z* amounts of F8-AZB and F10-AZB being populated, IR spectroscopic measurements were performed. IR data of F8-AZB_*x*_@DMOF-1 and F10-AZB_*x*_@DMOF-1 samples were recorded before and after irradiation with violet and green light, respectively. In Figure 3a,b, the IR characteristics of F8-AZB@DMOF-1 and F10-AZB@DMOF-1, respectively, are depicted. IR spectra of F8/10-AZB_0.125_@DMOF-1 and F8/10-AZB_3_@DMOF-1 are shown in Figures S20 and S21 in the Supporting Information. In the following section, the analysis of the IR data will start with F8-AZB@DMOF-1.

As visible in the virtually identical patterns of the nonirradiated sample for F8-AZB@DMOF-1 (Figure 3, top, dashed black line) and after exposure to violet light (blue line), the maximum population of the *E* isomer is already present without any violet light exposure. This observation corresponds to the data obtained from UV/vis spectroscopy. By green light exposure, the vibrational mode at ν = 1255 cm^–1^ increases while those at 1284, 1181, 1036, and 956 cm^–1^ decrease. The first band originates from the *Z* isomer, and the latter bands, from the *E* isomer. Although these modulations are observed, the complete vanishing of the mentioned IR characteristics was not found, suggesting that an *E*/*Z* mixture is present under both violet and green light irradiation. Analogous investigations were performed on F10-AZB_*x*_@DMOF-1. In Figure 3, bottom, the IR characteristics of F10-AZB@DMOF-1 are shown. IR spectra of F10-AZB_0.125_@DMOF-1 and F10-AZB_3_@DMOF-1 can be found in Figure S21 in the Supporting Information. In comparison to the IR characteristics of the initial state (Figure 3, bottom, dashed black line), irradiation with violet light does not induce any change in the vibrational modes (Figure 3, bottom, blue line). Consequently, the maximum amount of the *E* isomer that can be generated is already present right after the synthesis. Upon subsequent exposure to green light, a new band appears at approximately ν = 1110 cm^–1^, which is due to the presence of *Z*-F10-AZB. Because this band is not observed for the initial state and upon violet light exposure, 100% *E* isomer is present for these states.

To quantify the switching processes of all F8-AZB_*x*_@DMOF-1 and F10-AZB_*x*_@DMOF-1 compounds, characteristic vibrational bands of the *E* and *Z* isomers were chosen, and their peak intensities were related to each other. For F8-AZB, the vibrational modes at 1284 and 1258 cm^–1^ were selected and belong to the *E* and *Z* isomers, respectively. For F10-AZB, the characteristic bands at 980 cm^–1^ (*E* isomer) and 1110 cm^–1^ (*Z* isomer) were related to each other. In Table 2, the *E*/*Z* ratios for each dilution are given both in the initial state and after irradiation. These data are compared to the *E*/*Z* ratios of F4-AZB_*x*_@DMOF-1^9^ obtained via liquid-state NMR spectroscopic measurements.

**Table 2:** *E*/*Z* Ratios of F8-AZB_*x*_@DMOF-1 and F10-AZB_*x*_@DMOF-1 in the Initial State and after Irradiation with 405 and 535 nm,^a^ Compared to F4-AZB_*x*_@DMOF-1,^9^ Which Has Been Reported Previously^b^

		F8-AZB_*x*_@DMOF-1	F10-AZB_*x*_@DMOF-1	F4-AZB_*x*_@DMOF-1^9^
*x* = 0.125	initial	59% *E*, 41% *Z*	62% *E*, 38% *Z*	100% *E*, 0% *Z*
	405 nm	59% *E*, 41% *Z*	62% *E*, 38% *Z*	100% *E*, 0% *Z*
	535 nm	56% *E*, 44% *Z*	57% *E*, 43% *Z*	38% *E*, 62% *Z*
*x* = 1	initial	69% *E*, 31% *Z*	100% *E*, 0% *Z*	100% *E*, 0% *Z*
	405 nm	67% *E*, 33% *Z*	100% *E*, 0% *Z*	100% *E*, 0% *Z*
	535 nm	44% *E*, 56% *Z*	80% *E*, 20% *Z*	37.7% *E*, 62.3% *Z*
*x* = 3/1.5^b^	initial	56% *E*, 44% *Z*	100% *E*, 0% *Z*	88.7% *E*, 11.3% *Z*
	405 nm	56% *E*, 44% *Z*	100% *E*, 0% *Z*	93% *E*, 7% *Z*
	535 nm	42% *E*, 58% *Z*	80% *E*, 20% *Z*	60.5% *E*, 39.5% *Z*

a Irradiation time: 5 min.

b *x* value for F4-AZB.

Embedding in DMOF-1 preserves the photochromic behavior of F8-AZB, which has already been traced by UV/vis spectroscopy. For the 0.125 dilution, an almost equal ratio of both isomers is present in the initial state, which does not change upon irradiation with 405 nm. Surprisingly, only a little more *Z*-F8-AZB is populated when being exposed to green light. However, it must be stated that only small changes in the IR spectrum are visible as a result of the very high dilution. Compared to both the 1:1 and the 3:1 ratios, the amount of *E* isomer is approximately 10% higher in the initial state and after irradiation with violet light for F8-AZB_1_@DMOF-1. Nevertheless, green light exposure results in almost identical *Z* amounts of 56 and 58% for F8-AZB_1_@DMOF-1 and F8-AZB_3_@DMOF-1, respectively. The ratios obtained, especially for F8-AZB_0.125_@DMOF-1, are comparable to a study by Qin and co-workers,^37^ who investigated the suitability of MOF thin films with fluorinated azobenzene groups as electronic noise. By irradiation with different light sources, the authors found *E*-rich (wavelength of the irradiation source: λ = 400 nm), *Z*-rich (wavelength of the irradiation source: λ = 535 nm), and mixed states (wavelength of the irradiation source: λ = 450 nm), for which the latter shows an isomer ratio like that of F8-AZB@DMOF-1. As expected from the irradiation wavelength (which addresses the formation of both the *E* and *Z* isomers), Qin and co-workers found a nearly equivalent *E*-to-*Z* ratio upon 450 nm exposure. For F8-AZB, however, the mixed states are not a result of the irradiation wavelength but of interactions with the MOF host. These interactions will be thoroughly discussed in the SCC DFTB MD simulation part.

In the case of F10-AZB, similar photochromic behavior is observed for F10-AZB_0.125_@DMOF-1. Here, a significantly higher amount of *Z* isomer (38%) is already found in the initial state, which does not decrease upon violet light irradiation and increases only a little upon green light exposure (43%). Again, these *E*/*Z* ratios might be the result of the high dilution and corresponding small changes in the IR signatures. On the contrary, both F10-AZB_1_@DMOF-1 and F10-AZB_3_@DMOF-1 show a 100% presence of the *E* isomer in the initial state and after irradiation with 405 nm, while 20% *Z*-F10-AZB is populated upon green light exposure. Here, a higher amount of embedded dye does not influence the optical characteristics at all. Therefore, spatial restrictions can be completely excluded because the *E*/*Z* isomerization is not sterically hindered. By comparing the *E*/*Z* ratios of the 1:1 and 3:1 compositions for both fluorinated AZBs, the amount being embedded does influence the starting amount of *E* isomer for F8-AZB but not for F10-AZB. In the case of F8-AZB_3_@DMOF-1, a 10% lower *E* population might be explained by stronger *Z*-F8-AZB–DMOF-1 interactions, which is perfectly in line with the results of the theoretical calculations discussed in the following section. By comparing the *E*/*Z* ratios of F8-AZB and F10-AZB to the results obtained for F4-AZB, when embedded in DMOF-1,^9^ the degree of fluorination does significantly impact the switching efficiency. For F4-AZB, a higher amount of the *Z* isomer can be populated, which can be explained by almost equal interactions between *E*- and *Z*-F4-AZB with the host framework. In that way, irradiation with violet or green light induces the maximum formation of each isomer.^9^

### Understanding Host–Guest Interactions of Fx-AZBs inside DMOF-1 via SCC DFTB MD Simulations

Like the previous study of *E*/*Z*-F4-AZB@DMOF-1, the host–guest interactions have been analyzed in terms of the time series of key distances observed along the DFTB MD simulations.^9^ Again, the centroids of the aromatic parts of AZB and BDC^2–^ residues were used, which were determined as the average position of the six corresponding carbon atoms. The previous study has shown that the *E* and *Z* conformers of F4-AZB display dramatically different interaction motifs. Because the *Z* conformer is more compact, the preferred mode of interaction was found to involve BDC^2–^ residues within the same unit cell of the host lattice, which form a square coordination cavity. Typically, the *Z* conformer binds to BDC^2–^ residues in a perpendicular arrangement. However, the interaction is not particularly stable, and the guest molecule was free to rotate within the binding cavity, thereby changing coordination sites along the 100 ps simulation trajectory. On the other hand, the extended structure of the *E* conformer resulted in a preferred interaction involving two separate BDC^2–^ residues associated with neighboring unit cells of the host lattice. Although the guest molecule was subjected to librational motion, it did not change interaction sites over the course of the simulation. However, a short instance of a T-stacking conformation between a hydrogen in the para position in one of the aromatic F4-AZB moieties and the aromatic ring of a BDC^2–^ unit was observed.

Figure 6 displays the associated time series of host–guest centroid–centroid distances in the case of the *Z* conformer of F8-AZB inside DMOF-1. The structure was not yet fully equilibrated at the beginning of the sampling phase, and an additional interval of 12.5 ps was required to establish a stable interaction motif. The screenshots shown in Figure 6b,c reveal an interaction motif similar to that in the *Z*-F4-AZB case. However, the presence of additional F atoms appears to prevent any further rotation within the square binding cavity as inferred from the corresponding time series, yielding an average centroid–centroid distance of 0.354 nm for the final configuration.

The structural analysis of the *E*-F8-AZB conformer shown in Figure 7 reveals a different interaction motif that agrees well with the results observed in the case of *E*-F4-AZB.^10^ Again, the extended conformation favors interaction with two BDC^2–^ residues separated by one unit cell. However, in contrast to the *E*-F4-AZB case, the final interaction motif was established only after approximately 75 ps of simulation time (12.5 ps of equilibration plus 62.5 ps in the sampling phase). As can be seen from screenshots S1 and S2 taken at 7 and 25 ps on the sampling trajectory (cf. Figure 7b,c), the guest molecule appears to be stuck in the square interaction cavity associated with a single unit cell in the early stages of the simulation. This implies that the additional fluorine atoms in *E*-F8-AZB greatly reduce the guest mobility inside the host structure. The average centroid–centroid distance in the final configuration was determined to be 0.379 nm.

**Figure 8:**
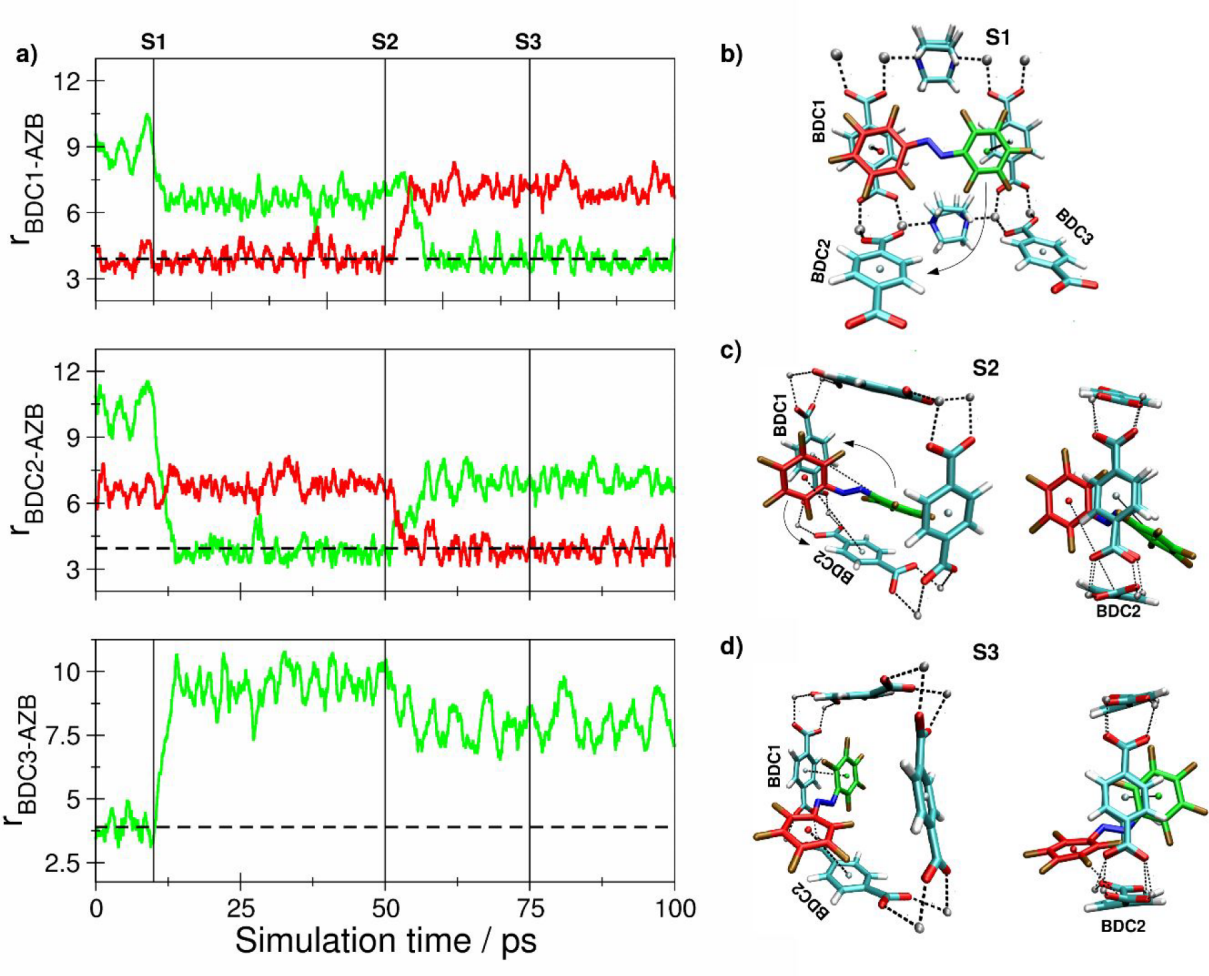
(a) Key distances between *E*-F10-AZB and BDC2^–^ groups of the DMOF-1 host structure based on the centroids of the aromatic units determined to be the average over the respective carbon atoms, resulting from a second MD simulation employing the final configuration of the *E*-F8-AZB simulation as a template to generate the initial structure. (b–d) Snapshots displaying representative configurations of the *E*-F10-AZB@DMOF-1 interaction taken from the simulation trajectory. The associated time steps are marked as S1 to S3 in the time series.

The *Z* conformer of F10-AZB shows an interaction motif similar to that previously observed for *Z*-F4-AZB and *Z*-F8-AZB (cf. Figure 8). However, because the ligand is even larger in this case, it required approximately 60 ps (12.5 ps of equilibration plus 57.5 ps of sampling) to establish the preferred interaction motif, which persisted until the end of the simulation. However, as shown by the side view of each configuration in the screenshots in Figure 8c,d, the ligand cannot fully occupy the coordination cavity formed by the four adjacent BDC^2–^ units. Consequently, the interaction motif is distorted, with the aromatic rings being slightly dislocated from the cavity, resulting in a notable increase in the average centroid–centroid distance of 0.369 nm compared to the value of 0.354 nm in the case of *Z*-F8-AZB@DMOF-1.

**Figure 9:**
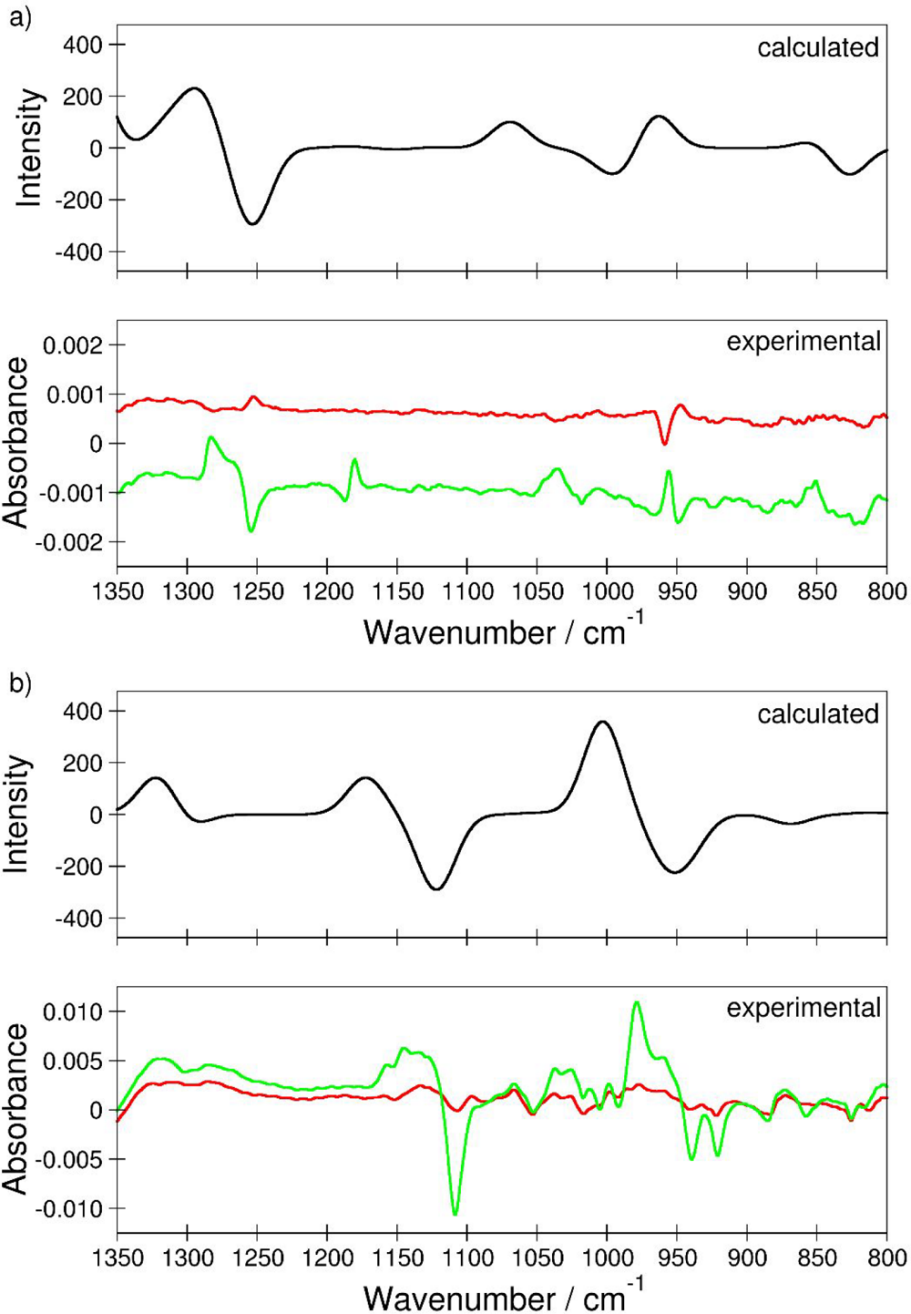
Comparison of the calculated difference spectra determined from the scaled harmonic frequencies obtained at the B3LYP-GD3BJ/6-31G(d,p) level (black) for (a) *E*/*Z*-F8-AZB and (b) *E*/*Z*-F10-AZB with their experimental counterparts measured after irradiation at 405 nm (red) and 535 nm (green).

Until this point, the structural properties observed in the simulations of the host–guest complexes were essentially in agreement with the results reported earlier for *E*/*Z*-F4-AZB@DMOF-1.^9^ However, in the case of *E*-F10-AZB, a different situation is observed. As seen from the associated distance plots and representative screenshots shown in Figure 9, the conformer remains within the square coordination cavity associated with a single unit cell; i.e., the extended coordination motif involving BDC^2–^ residues of two different unit cells as observed for *E*-F4- and *E*-F8-AZB has not been formed.

**Figure 10:**
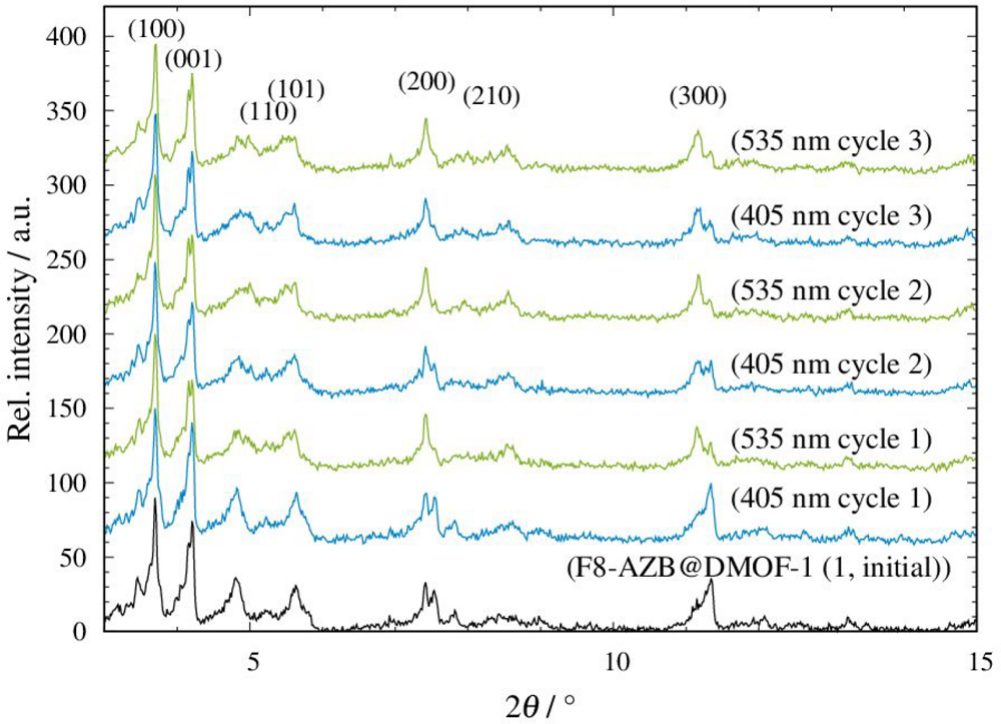
F8-AZB@DMOF-1 before (black line) and after irradiation with violet (blue lines) and green light (green lines). Three switching cycles were performed. The diffraction patterns were measured at 298 K (Stoe Stadi P: λ = 0.7093 Å).

Although the phenyl rings of *E*-F10-AZB are interacting with the aromatic rings of the involved BDC^2–^ moieties, it is the azo group of the guest molecule that remains confined in the coordination cavity (screenshot S2). Therefore, this system shows a significantly increased average centroid–centroid distance of 0.395 nm as evaluated for the final configuration. During the simulation, the guest molecule did not show any attempt to establish a different coordination motif. It was therefore of interest to investigate whether a poorly chosen initial structure is responsible for this deviant result, and a second DFTB MD simulation of *E*-F10-AZB@DMOF-1 was performed (cf. Figure 10).

In this case, the final configuration of the *E*-F8-AZB system was employed as a template to generate the initial configuration; i.e., the *E*-F10-AZB molecule was explicitly set into the extended configuration bound to BDC^2–^ residues associated with two different unit cells (cf. Figure 7d). Although this configuration remained for a total simulation time of about 22.5 ps (12.5 ps of equilibration plus 10 ps of sampling), the structure was subjected to several rearrangements. In the final structure, the same coordination as observed in the first simulation of *E*-F10-AZB@DMOF-1 was again established after a total simulation time of approximately 65 ps. A similar average centroid–centroid distance of 0.392 nm, in good agreement with the first simulation, was observed. Realizing that the second DFTB MD simulation resulted in the same coordination motif despite starting from an entirely different initial configuration confirms that the *E*-F10-AZB molecule is a distinct guest among all investigated species. The fact that this molecule displays a different interaction with the DMOF-1 host structure agrees well with the experimental observations, showing that the *E* isomer is more stabilized within the DMOF-1 host than the *Z* isomer, which can be generated only to 20% upon irradiation with green light.

To characterize the interaction in more detail, the instantaneous host–guest interaction potentials have been evaluated, and the respective time series are shown in Figure S22 in the Supporting Information. From these, the average interaction potentials ⟨*U*_int_⟩ were determined by averaging over the last 25 ps of each simulation. In both cases, the *Z* conformer displays a more favorable interaction compared to its *E* counterpart. The respective difference amounts to 34.1 and 11.1 kJ·mol^–1^ in the cases of F8- and F10-AZB, respectively, which is in strong contrast to the previous investigations on F4-AZB, in which a difference of just 0.9 kJ·mol^–1^ in favor of the *Z* conformer was observed. The analysis of the average binding potential shows that despite the differences in the binding motif found for the three investigated AZB derivatives, the F8-AZB molecule does not switch as efficiently as the other investigated fluorinated azobenzenes, which is also evident in the *E*/*Z* ratios after violet and green light irradiation, respectively (Table 2). On the contrary, 100% *E* isomer is present in the initial state and after irradiation with violet light for F10-AZB@DMOF-1 and F10-AZB_3_@DMOF-1 (Table 2). In particular, the comparably larger energy difference of 34.1 kJ·mol^–1^ between the *Z* and *E* conformers of F8-AZB provides a direct explanation of the shift in the initial distribution in favor of the *Z* isomer.

It should be noted at this point that there are no corresponding differences in the energies of the individual *E*/*Z* conformers but there is an average interaction energy between the guest molecule and the host structure. While the energy differences are comparably large, they are small compared to the energy associated with the change from the ground state to the electronically excited state. In this work, irradiation at 405 and 535 nm is employed, corresponding to excitation energies of 295 and 223 kJ·mol^–1^, respectively. Thus, the energies required to reach the electronically excited state are almost 1 order of magnitude larger compared to the difference in the guest–host interaction energy determined for the different conformers in the electronic ground state. However, although irradiation enables the excitation of the electronic structure, it does not necessarily imply that a conformational change is successful. When a particular interaction between the MOF host structure and the photoswitch prevents a conformational change, e.g., if the conformer is strongly bound to the structure of the host system, then the switch efficiency might be greatly impeded. For this reason, the MD simulations have been carried out to identify the preferred interaction sites in the host structure.

### Vibrational Difference Spectra

The conclusions drawn from the DFTB MD simulations are further emphasized by the analysis of the experimental vibrational difference spectra, which were compared to their theoretically calculated counterparts. In a previous study on fulgide@MOF,^18^ a similar analysis enabled the discrimination of the photoswitching mechanism by comparing the differences in the vibrational spectra obtained for the initial state prior to irradiation with those obtained for two potential reaction products. Figure 9 depicts a comparison of the theoretically determined vibrational difference spectra obtained for the *E* → *Z* transition of F8- and F10-AZB in comparison to the experimental results determined as the difference in the initial state and measurements obtained after irradiation at 405 and 535 nm, respectively (Figure 3, top and bottom, and Figures S20 and S21, Supporting Information).

The difference in absorbance between the experimental difference spectra, being a factor of 5 lower in the case of F8-AZB (Figure 9a), should be explicitly highlighted at this point, indicating a dramatically reduced switching efficiency. Despite these differences, the spectral data clearly show that no switching occurred upon irradiation at 405 nm. On the other hand, the characteristic pattern observed in the theoretically calculated difference spectra is clearly visible after irradiation of the samples at 535 nm. This data confirms an improved switching efficiency of fully fluorinated compound F10-AZB compared to F8-AZB and is in line with the experimentally determined *E*/*Z* ratios (Table 2).

### Tracing-Light-Induced Structural Changes in Fx@AZB@DMOF-1 Systems

In addition to tracing the switching efficiency of F8-AZB and F10-AZB inside DMOF-1, potential light-induced guest-to-host structural transmissions were studied. In a previous publication by Kitagawa and co-workers on plain azobenzene inside DMOF-1,^4^ light exposure resulted in *E*-to-*Z* isomerization on the inserted dye. The structural rearrangement of AZB was reversibly transmitted to the host structure. On the contrary, only minor structural changes in the host structure were observed upon *E*-to-*Z* conversion of the inserted *ortho*-tetrafluorinated azobenzene (F4-AZB).^9^ This was surprising because the isomerization process of the *E* and *Z* isomer of F4-AZB is much more efficient than that of plain nonfluorinated azobenzene. Consequently, interactions between DMOF-1 and azobenzene were assumed to be stronger than those between the host matrix and F4-AZB, which was further confirmed by IR spectroscopic measurements and complementary molecular dynamics simulations. Because of the significantly higher switching yields of F8-AZB and F10-AZB dissolved in acetonitrile,^34^ the light-induced *E*-to-*Z* conversion was assumed to impact the overall DMOF-1 host structure in a more efficient way compared to the impact on AZB and F4-AZB. To account for both (i) the loading amount and (ii) the degree of fluorination, possible light-induced structural changes were studied as a function of these parameters for F8-AZB and F10-AZB.

DMOF-1 belongs to the class of MOFs that show the “breathing effect”^38^ upon guest enclosure: the framework is not rigid but flexible and accordingly responds to attractive and repulsive interactions with structural changes. This response is visible in the positional changes of reflections and the appearance of new reflections in the diffraction pattern. Consequently, F8-AZB and F10-AZB insertion causes such changes in the diffraction characteristics (Figures S5 and S6, Supporting Information). Additionally, light-induced *E*-to-*Z* conversion changes the binding motifs of both guests inside DMOF-1, which has been shown in detail by DFTB MD simulations. These alterations in binding motifs were expected to impact the overall diffraction characteristics of DMOF-1. The respective XRPD patterns are depicted in Figure 10 and Figures S23 and S24 in the Supporting Information.

At guest-to-host molar ratios of 0.125:1 and 3:1, irradiation with violet and green light does not cause any significant change in the reflection patterns of F8-AZB@DMOF-1 systems (Figure S23, top and bottom, Supporting Information). Equal molar ratios, however, lead to small reflection intensity modulations: the reflection at approximately 7.5° (2θ) shows a splitting in the initial state, which remains upon violet light exposure (Figure 10). Green light irradiation causes a decrease in the right splitting part, and the left gains in intensity. This process is not reversible over three switching cycles. The same accounts for the reflection at approximately 11.25° (2θ). Here, reflection splitting is observed upon green light exposure. Nevertheless, the original pattern is not retained after three switching cycles. By comparing the reflection patterns of the F10-AZB_*x*_@DMOF-1 composite materials before and after violet and green light exposure, similar characteristics were found. The respective diffraction patterns are depicted in Figure S25 in the Supporting Information. Here, for loading amounts of 0.125 and 3 (Figure S24, top and bottom, Supporting Information), violet and green light exposure does not cause any change in the diffraction patterns, whereas analogous reflection modulations as already described for equal molar ratios of F8-AZB to DMOF-1 are found for F10-AZB@DMOF-1 (Figure S24, center, Supporting Information). Comparing the results obtained to previously reported observations on F4-AZB,^9^ the degree of fluorination does not have any impact on the host structure. Moreover, the loading amount contributes very little to possible structural changes of the host matrix. With 0.125 Fx-AZB inside DMOF-1, host–guest interactions are probably too small, and dense packing for a large amount such as 3 Fx-AZB may limit mobility. In equal molar ratios, few interactions occur for both F8-AZB and F10-AZB inside DMOF-1, which is visible in small reflection modulations that do not seem to be reversible. Conclusively, the expected impact of the *E*–*Z* conversion of F8-AZB and F10-AZB on host structure DMOF-1 was not found because it had previously been reported for nonsubstituted AZB. By the introduction of fluorine atoms, the switching efficiency is increased in solution, but not when embedded in DMOF-1.

## Conclusions

Within this fundamental study, we further contribute to the understanding of the impact of single components of switch@MOF systems on the overall materials’ optical characteristics. Finding ways to control those as a function of dye substitution and loading amount is an elegant way to systematically design functional materials with the long-term perspective of being implemented in data storage devices. By varying the degree of fluorination, the emerging photochromic response of the inserted Fx-AZBs (F8-AZB and F10-AZB) directly reflects the stabilization effects of the *E* and *Z* isomers within the framework as a function of the physicochemical properties of the dye molecule. Furthermore, possible guest–guest interactions are considered by varying the amount of Fx-AZB per MOF pore. All F8-AZB_*x*_@DMOF-1 and F10-AZB_*x*_@DMOF-1 systems show a reversible photochromic response upon violet and green light irradiation without any hint of fatigue. Because of the few host–guest interactions determined via IR spectroscopy, the *E*-to-*Z* conversion hardly causes any structural changes to the flexible host framework. Interestingly, for F8-AZB_*x*_@DMOF-1, a significantly reduced switching efficiency was found to be almost independent of *x*, whereas full fluorination in the case of the F10-AZB_*x*_@DMOF-1 systems caused a tremendous stabilization of the *E* isomer, again independent of *x*.

The observed differences in the switching efficiencies between the investigated systems have been further studied with SCC DFTB MD simulations of the different guest molecules in both the *E* and *Z* conformations inside DMOF-1, yielding manifold insights into the structural and dynamic properties associated with the respective interaction motifs. In the cases of *E*/*Z*-F8-AZB and *Z*-F10-AZB, host–guest interactions similar to those previously reported for *E*/*Z*-F4-AZB^10^ were observed, and the *E* conformer of F10-AZB displayed a dramatically different interaction motif. Instead of binding to BDC^2–^ residues associated with two different unit cells of the host structure, *E*-F10-AZB remains confined to the square coordination cavity formed by four perpendicularly arranged BDC^2–^ moieties of a single unit cell. These unexpected results were confirmed in a second DFTB MD simulation that explicitly assumed the extended binding motif observed for *E*-F8-AZB. Also in this case, the previously observed coordination was formed within the simulation time. Despite this difference in the interaction motif, F8-AZB shows the largest difference in the average interaction energy when switching between the *E* and *Z* conformers.

As outlined in the Results section, the determined values for Δ*U*_int_ measure the strength of interaction between the respective conformer and the host structure. To induce the photoswitching reaction, the guest molecule has to (at least partially) desorb from its interaction site to provide access for the conformational reaction path. In the case of F8-AZB, the MD simulations show that the interaction motifs of the *E* and *Z* conformers are entirely different, requiring the migration of the molecule throughout the MOF structure. In contrast, in the case of the F10-AZB molecule, both conformers share the same interaction site within the host. On the basis of these differences observed for F8- and F10-AZB in the MD simulations, it can be expected that the *Z*-to-*E* conversion is less favorable in the F8-AZB case, whereas it can be carried out with higher efficiency for F10-AZB. This is exactly the case in the experimental observation, yielding an *E*/*Z* ratio of 1:1 for F8-AZB, while the ratio is strongly shifted toward the *E* conformer in the F10-AZB case by about a factor of 4, i.e., a ratio of 2:8.

With this study, we further deepen the insight into the interplay between the single components of guest–host systems. Understanding those interactions as a function of the local environment and dye substitution is indispensable to systematically designing responsive functional materials with technologically relevant characteristics. Knowing the exact adjusting screws combined with their resulting impact on the overall material will pave the way to new systems with desirable properties.

## Supporting Information Available

NMR spectra of F8- and F10-AZB; XRPD patterns of the Fx-AZB@DMOF-1 systems and unloaded DMOF-1; ^1^H NMR spectra of F8-AZB_*x*_@DMOF-1; additional UV/vis and IR spectra of F8-/F10-AZB_*x*_@DMOF-1; time series of the instantaneous interaction potential Δ*U*_in__t_; detailed listing of composition calculations on F8/F10-AZB@DMOF-1 systems; and equilibrium geometries and unscaled vibrational frequencies ν_calc_ of F8- and F10-AZB (PDF)
