# Supplementary material for: Fundamental Study of the Optical and Vibrational Properties of Fx-AZB@MOF systems as Functions of Dye Substitution and the Loading Amount
Source: Langmuir. 2022 Mar 28;38(14):4295–309. doi: 10.1021/acs.langmuir.1c03482 (PMC9009183; doi:10.1021/acs.langmuir.1c03482)
Supplement: Supplementary file 1 — la1c03482_si_001.pdf [file la1c03482_si_001.pdf]

Supporting Information

Fundamental study on the optical and vibrational properties of Fx-AZB@MOF systems as a function of dye substitution and loading amount

Markus Rödl[a], Alen Reka[a], Marko Panic[a], Alexander Fischereder[a], Marco Oberlechner[a], Thomas Mairegger[a], Holger Kopacka[a], Hubert Huppertz[a], Thomas S. Hofer[a,*], and Heidi A. Schwartz[a,*]

[a]*Institute of General, Inorganic and Theoretical Chemistry, University of Innsbruck, Innrain 80-82, A-6020 Innsbruck, Austria.*

Corresponding author main: Heidi A. Schwartz

E-mail: heidi.schwartz@uibk.ac.at

Corresponding author theory: Thomas S. Hofer

E-mail: t.hofer@uibk.ac.at

Content

Figure S1 and S2. ^{1}H and ^{19}F NMR spectra of F8-AZB.

Figure S3. ^{19}F NMR spectra of F10-AZB.

Figure S4. Diffraction pattern of unloaded DMOF-1 in comparison to the peak intensities and position calculated from theoretical data.

Figure S5 to S6. XRPD patterns of F8$_x$-AZB@DMOF-1 and F10$_x$-AZB@DMOF-1 compared to unloaded DMOF-1.

Figure S7 to S12. IR spectra of F8/F10-AZB$_x$@DMOF-1 systems before and after irradiation with violet and green light in comparison to non-loaded DMOF-1.

Figure S13 to S15. ^{1}H NMR spectra of F8-AZB$_x$@DMOF-1.

Figure S16. UV/Vis reflectance spectra of F8-AZB@DMOF-1 and F10-AZB@DMOF-1 before and after irradiation with violet light over a total of 5 min.

Figure S17. UV/Vis reflectance spectra of F8-AZB@DMOF-1 and F8-AZB$_3$@DMOF-1 before and after irradiation with violet and green light over ten switching cycles.

Figure S18. UV/Vis reflectance spectra of F10-AZB$_{0.125}$@DMOF-1 before and after irradiation with violet and green light and over ten switching cycles.

Figure S19. UV/Vis reflectance spectra of F10-AZB@DMOF-1 and F10-AZB$_3$@DMOF-1 before and after irradiation with violet and green light over ten switching cycles.

Figure S20. IR spectra of F8-AZB$_{0.125}$@DMOF-1 and F8-AZB$_3$@DMOF-1 before and after irradiation with violet and green light.

Figure S21. IR spectra of F10-$AZB_{0.125}$@DMOF-1 and F10-AZB_3@DMOF-1 before and after irradiation with violet and green light.

Figure S22. Time series of the instantaneous interaction potential ΔU_{int}

Figure S23. Diffraction patterns of F8-$AZB_{0.125}$@DMOF-1 and F8-AZB_3@DMOF-1 before and after irradiation with violet and green light. Three switching cycles were performed.

Figure S24. Diffraction patterns of F10-$AZB_{0.125}$@DMOF-1, F10-AZB@DMOF-1 and F10-AZB_3@DMOF-1 before and after irradiation with violet and green light. Three switching cycles were performed.

Tables S1-S8. Equilibrium geometries as well as the unscaled vibrational frequencies υ_{calc} of F8- and F10-AZB.

Tables S9 and S10. Detailed listing of composition calculations on F8/F10-AZB@DMOF-1 systems.

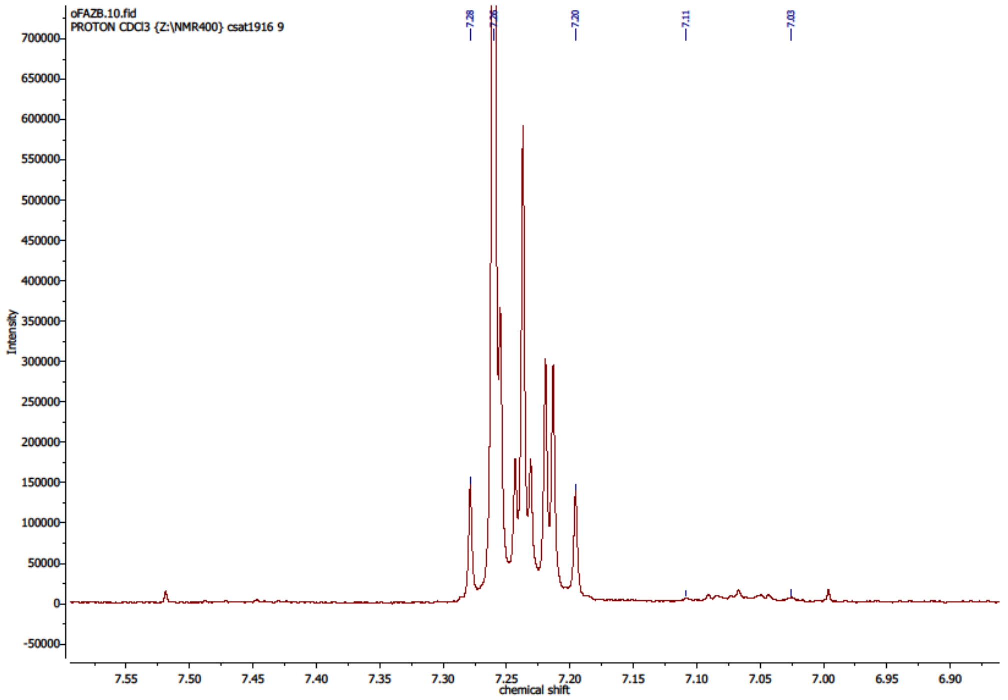

Figure S1. ^{1}H NMR spectrum of F8-AZB dissolved in $CDCl_3$.

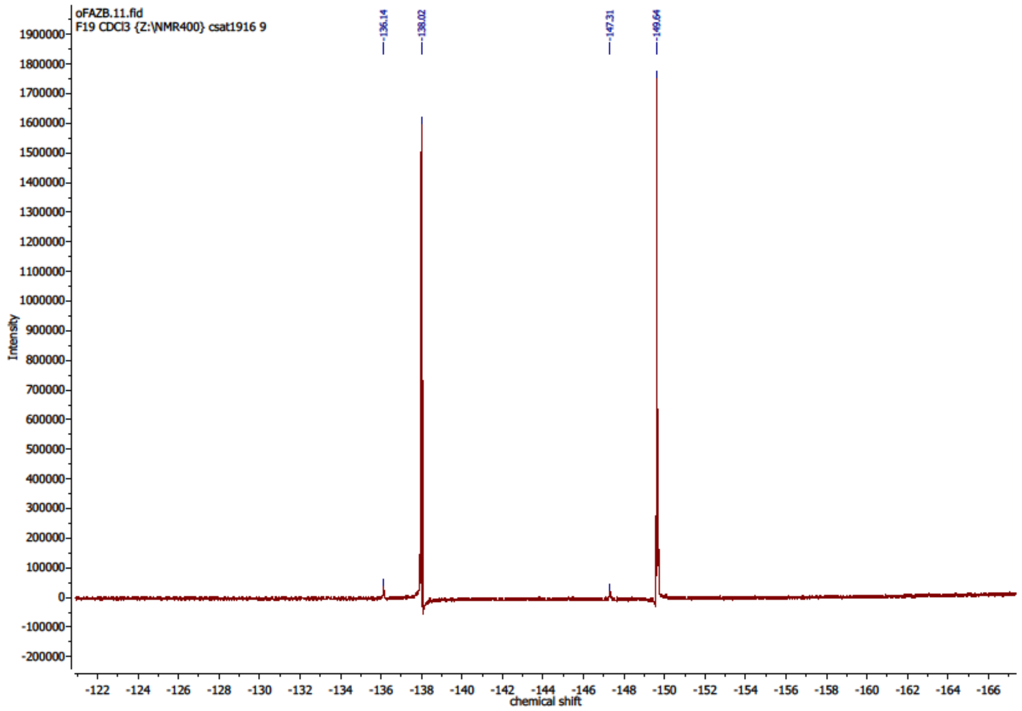

Figure S2. ^{19}F NMR spectrum of F8-AZB dissolved in $CDCl_3$.

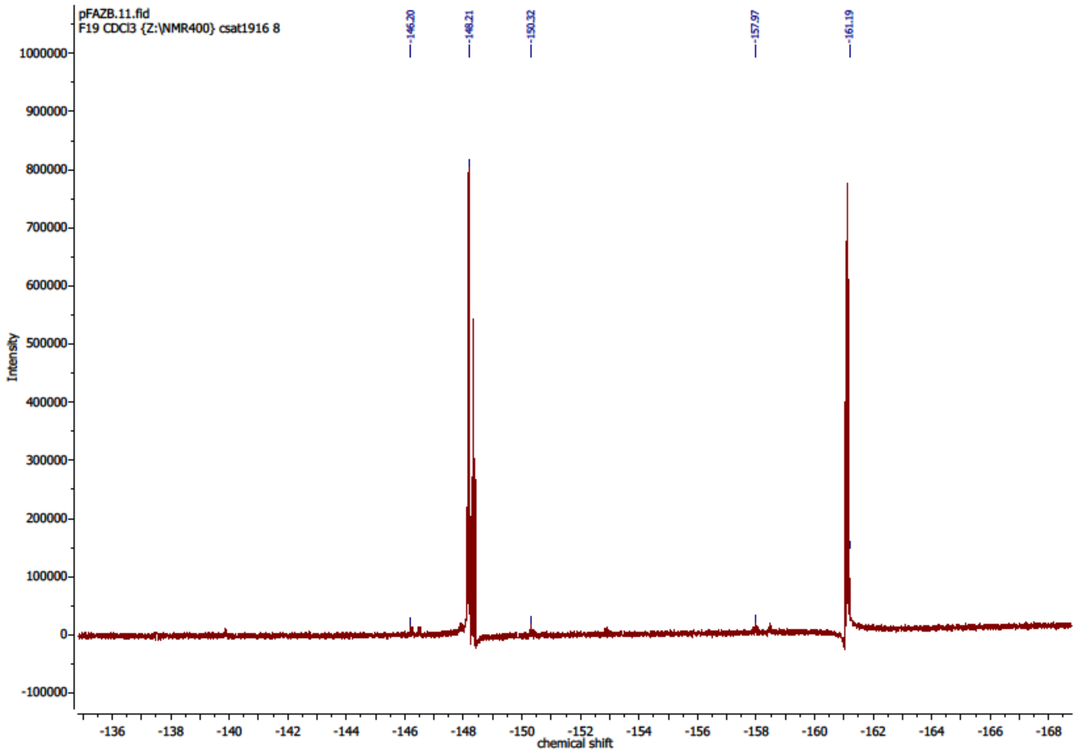

Figure S3. ^{19}F NMR spectrum of F10-AZB dissolved in $CDCl_3$.

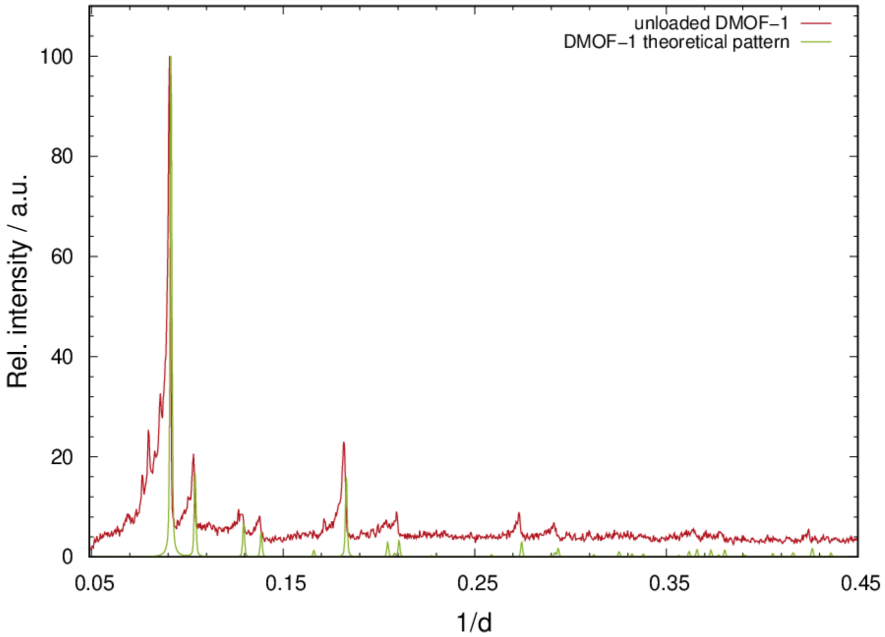

Figure S4. XRPD patterns of non-loaded DMOF-1 (red line), and theoretical data of non-loaded DMOF-1 (light green line). The diffraction pattern was measured at 298 K (*Stoe Stadi P*: λ = 0.7093 Å).

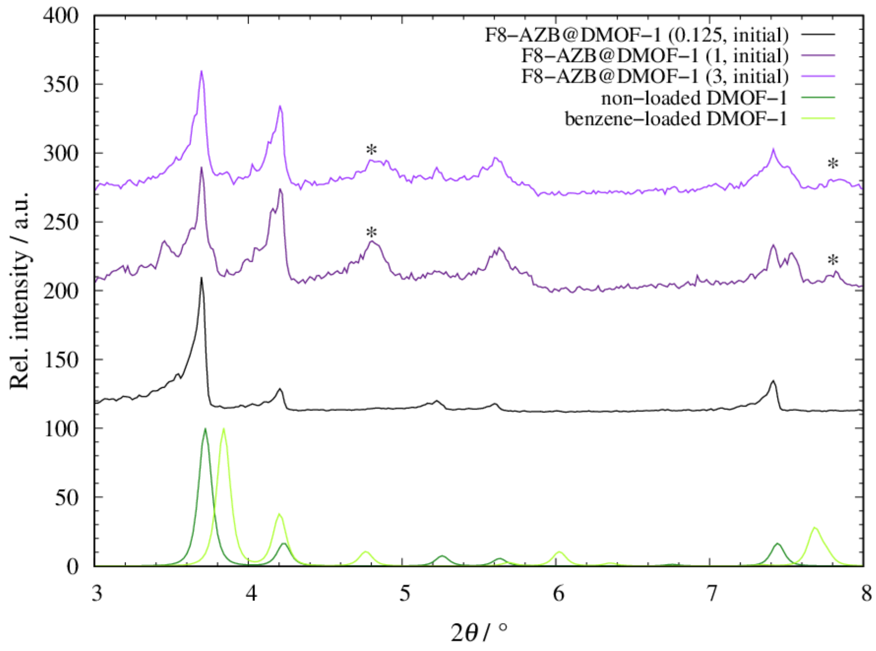

Figure S5. Diffraction patterns F8$_x$-AZB@DMOF-1 (dark violet, violet and violet line), non-loaded DMOF-1 (dark green line), and benzene-loaded DMOF-1 (light green line). The diffraction patterns were measured at 298 K (*Stoe Stadi P*: λ = 0.7093 Å).

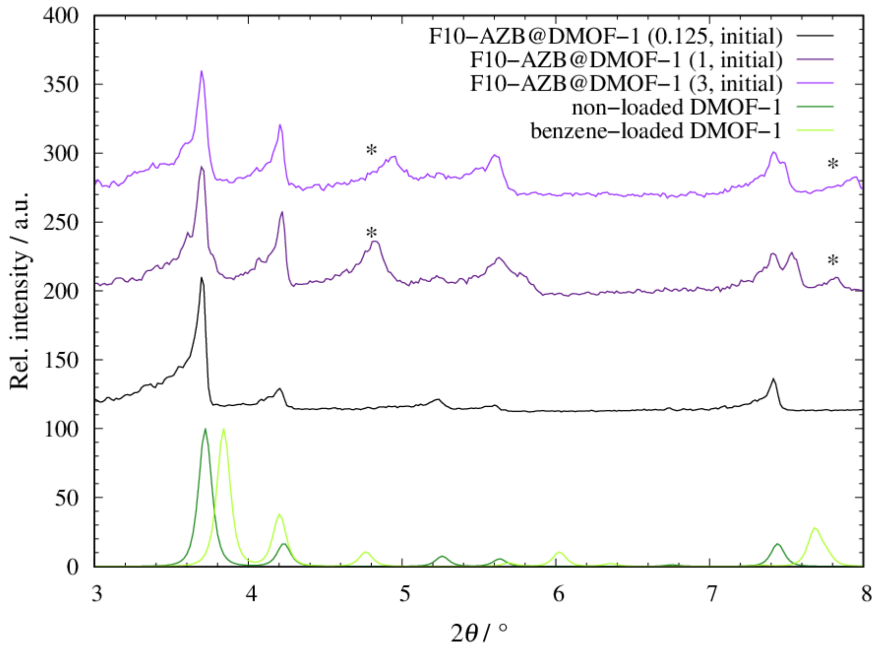

Figure S6. XRPD patterns F10$_x$-AZB@DMOF-1 (dark violet, violet, and violet line), non-loaded DMOF-1 (dark green line), and benzene-loaded DMOF-1 (light green line). The diffraction patterns were measured at 298 K (*Stoe Stadi P*: λ = 0.7093 Å).

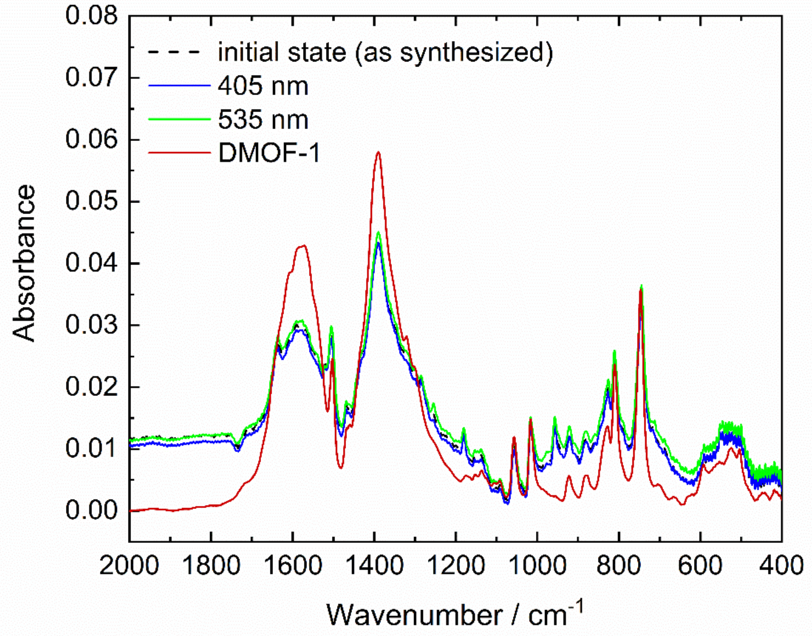

Figure S7. IR spectra of F8-AZB$_{0.125}$@DMOF-1 before (dashed black line) and after irradiation with violet (blue line) and green light (green light) in comparison to non-loaded DMOF-1 (red line).

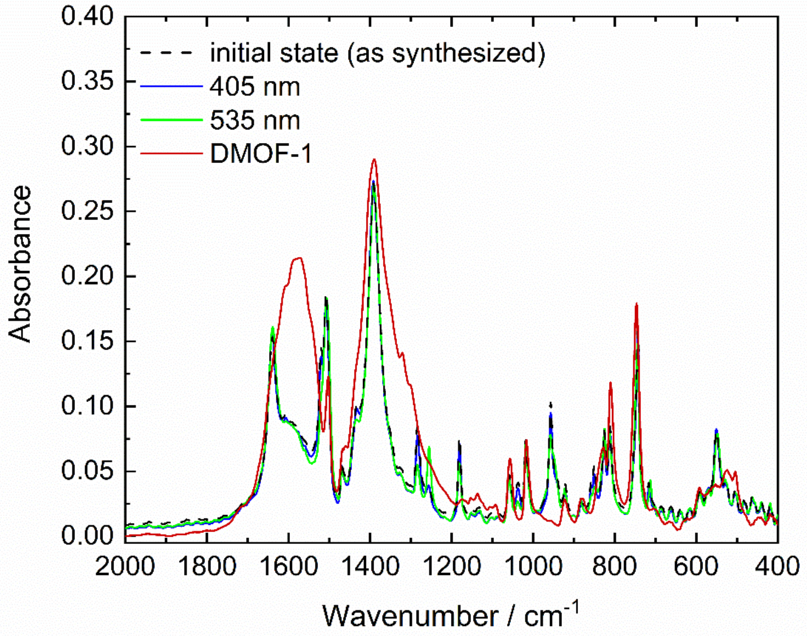

Figure S8. IR spectra of F8-AZB$_{1}$@DMOF-1 before (dashed black line) and after irradiation with violet (blue line) and green light (green light) in comparison to non-loaded DMOF-1 (red line).

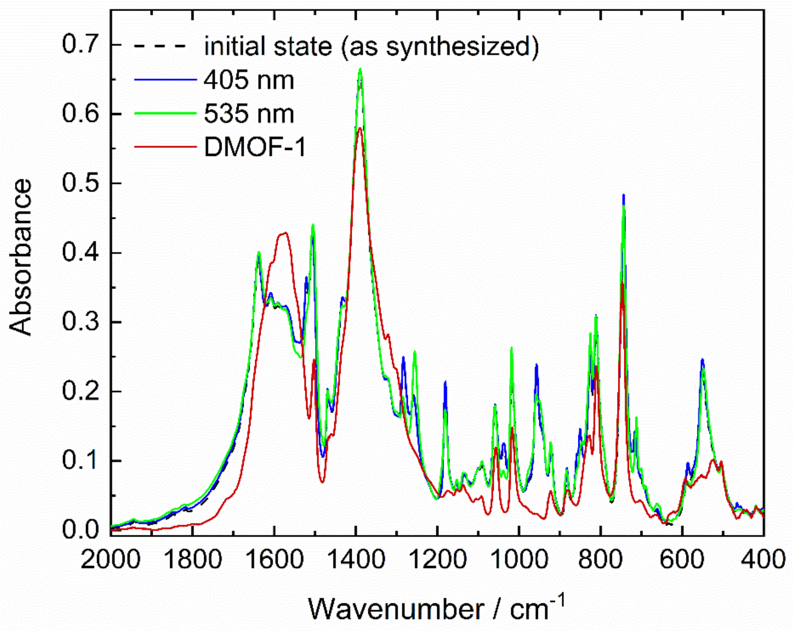

Figure S9. IR spectra of F8-AZB$_3$@DMOF-1 before (dashed black line) and after irradiation with violet (blue line) and green light (green light) in comparison to non-loaded DMOF-1 (red line).

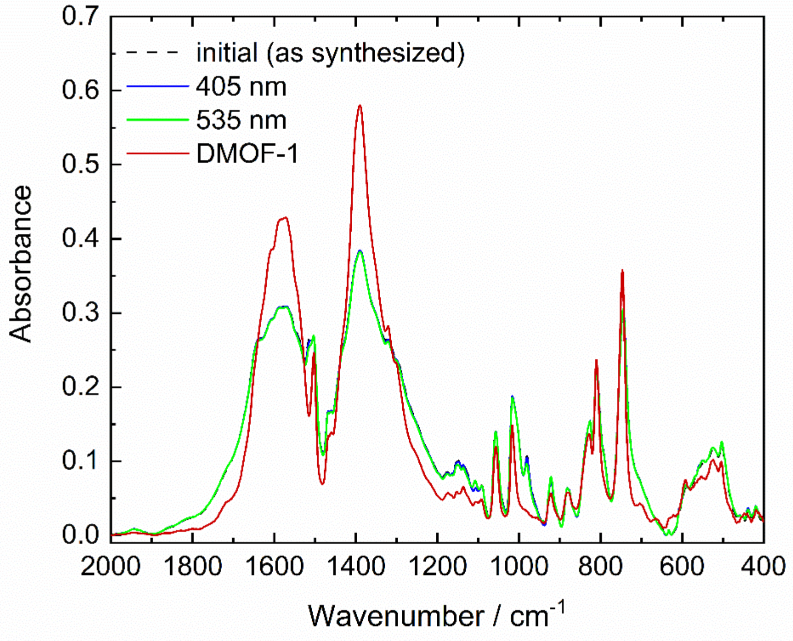

Figure S10. IR spectra of F10-AZB$_{0.125}$@DMOF-1 before (dashed black line) and after irradiation with violet (blue line) and green light (green light) in comparison to non-loaded DMOF-1 (red line).

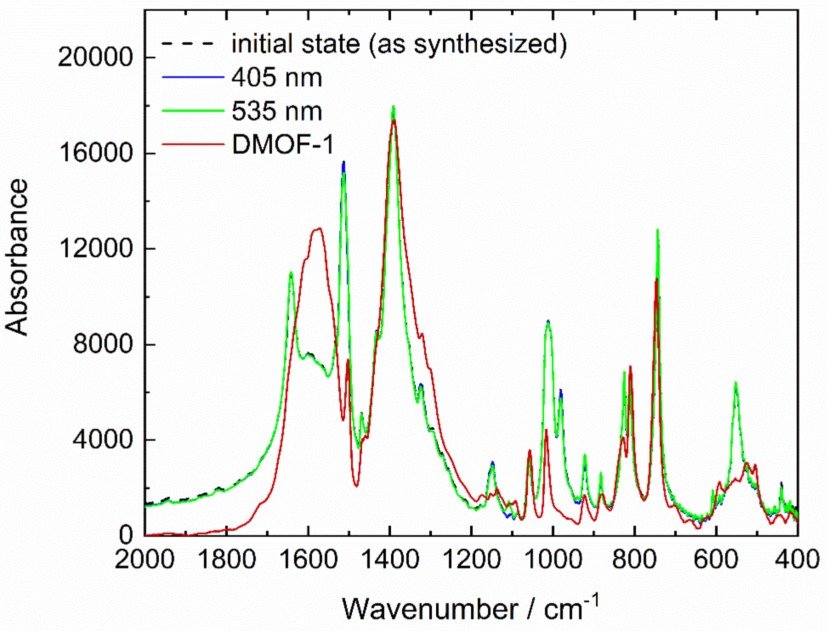

Figure S11. IR spectra of F10-AZB$_1$@DMOF-1 before (dashed black line) and after irradiation with violet (blue line) and green light (green light) in comparison to non-loaded DMOF-1 (red line).

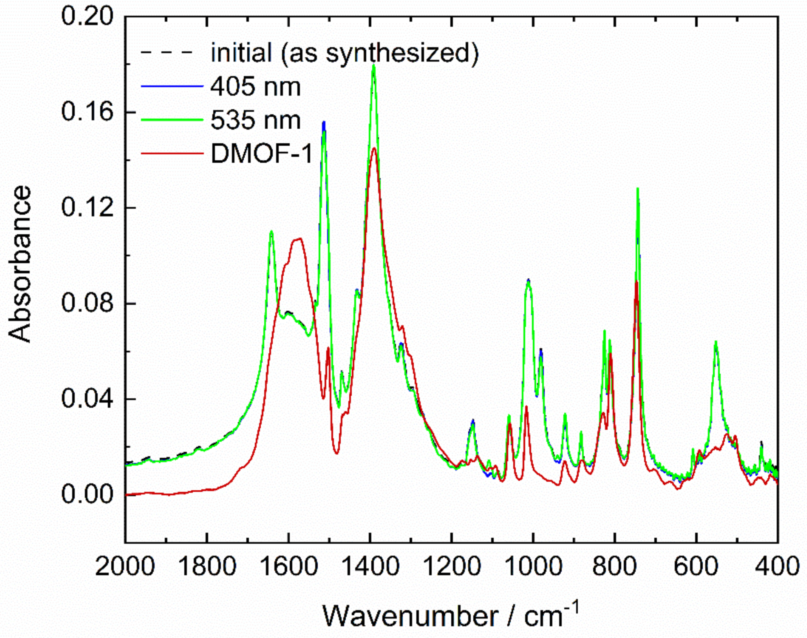

Figure S12. IR spectra of F10-AZB$_3$@DMOF-1 before (dashed black line) and after irradiation with violet (blue line) and green light (green light) in comparison to non-loaded DMOF-1 (red line).

Table S1. Minimum configuration of the *Z* conformer of F8-AZB obtained at B3LYP-GD3BJ/6-31G(d,p) level in implicit solvation (ε=24.852).

N	-0.618265	0.089665	-2.128926
N	0.618266	-0.089663	-2.128926
C	-1.343095	0.302716	-0.916489
C	-1.035693	1.331170	-0.020465
F	0.050838	2.096384	-0.213491
C	-1.867423	1.589473	1.062713
F	-1.536936	2.587711	1.897679
C	-3.014846	0.842048	1.284511
H	-3.653613	1.044774	2.135891
C	-3.323142	-0.172715	0.388172
F	-4.420715	-0.924303	0.568354
C	-2.514493	-0.429192	-0.710876
F	-2.829948	-1.417358	-1.559077
C	1.343097	-0.302724	-0.916494
C	2.514494	0.429188	-0.710879
F	2.829943	1.417360	-1.559076
C	3.323141	0.172717	0.388171
F	4.420712	0.924307	0.568351
C	3.014845	-0.842043	1.284513
H	3.653610	-1.044767	2.135896
C	1.867424	-1.589471	1.062715
F	1.536939	-2.587710	1.897681
C	1.035693	-1.331171	-0.020463
F	-0.050835	-2.096390	-0.213489

Table S2. Minimum configuration of the *E* conformer of F8-AZB obtained at B3LYP-GD3BJ/6-31G(d,p) level in implicit solvation (ε=24.852).

N	0.409900	-0.482156	-0.038130
N	-0.409898	0.482147	-0.038134
C	1.765552	-0.120327	-0.022999
C	2.323618	1.164856	0.138425
F	1.560386	2.251565	0.292806
C	3.702359	1.335209	0.159208
F	4.185796	2.578700	0.320366
C	4.576298	0.265609	0.019888
H	5.648847	0.419101	0.034971
C	4.037368	-1.001761	-0.138956
F	4.840876	-2.068805	-0.278460
C	2.663003	-1.197359	-0.155879
F	2.191744	-2.440287	-0.308728
C	-1.765551	0.120322	-0.023000
C	-2.662998	1.197358	-0.155881
F	-2.191735	2.440284	-0.308730
C	-4.037364	1.001765	-0.138956
F	-4.840868	2.068813	-0.278460
C	-4.576299	-0.265603	0.019887
H	-5.648848	-0.419091	0.034971
C	-3.702364	-1.335206	0.159208
F	-4.185806	-2.578695	0.320365
C	-2.323622	-1.164858	0.138425
F	-1.560395	-2.251571	0.292805

Table S3. Minimum configuration of the Z conformer of F10-AZB obtained at B3LYP-GD3BJ/6-31G(d,p) level in implicit solvation (ε=24.852).

N	0.623814	-2.345767	0.042839
N	-0.623816	-2.345728	-0.044641
C	1.363246	-1.134924	0.202116

C	1.120735	-0.219862	1.232131
F	0.085810	-0.393512	2.065924
C	1.957538	0.870082	1.432493
F	1.712545	1.733167	2.424332
C	3.060513	1.056749	0.605211
F	3.862011	2.107127	0.789831
C	3.326347	0.149922	-0.417643
F	4.382852	0.335982	-1.215799
C	2.495741	-0.947001	-0.594906
F	2.755581	-1.809887	-1.582731
C	-1.363241	-1.134754	-0.202973
C	-2.495746	-0.947451	0.594178
F	-2.755596	-1.811100	1.581333
C	-3.326360	0.149600	0.417743
F	-4.382878	0.335041	1.216025
C	-3.060523	1.057209	-0.604416
F	-3.862032	2.107717	-0.788247
C	-1.957525	0.871197	-1.431813
F	-1.712520	1.735050	-2.422981
C	-1.120718	-0.218895	-1.232276
F	-0.085775	-0.391894	-2.066183

Table S4. Minimum configuration of the *E* conformer of F10-AZB obtained at B3LYP-GD3BJ/6-31G(d,p) level in implicit solvation (ε=24.852).

N	-0.399238	-0.491557	0.046066
N	0.399241	0.491578	0.046019
C	-1.760829	-0.162279	0.030645
C	-2.350294	1.111295	-0.116006
F	-1.615213	2.216620	-0.251901
C	-3.729613	1.263472	-0.141671
F	-4.266730	2.480782	-0.286434
C	-4.563222	0.154840	-0.020712
F	-5.886453	0.312040	-0.043843
C	-4.012895	-1.113538	0.126456
F	-4.811424	-2.180155	0.246624
C	-2.634145	-1.261884	0.145389
F	-2.136743	-2.493500	0.283696
C	1.760831	0.162291	0.030631
C	2.634154	1.261890	0.145376
F	2.136761	2.493510	0.283678
C	4.012903	1.113532	0.126456
F	4.811440	2.180142	0.246632
C	4.563221	-0.154851	-0.020707
F	5.886451	-0.312062	-0.043828
C	3.729604	-1.263476	-0.141673
F	4.266712	-2.480790	-0.286436
C	2.350286	-1.111287	-0.116015
F	1.615195	-2.216604	-0.251925

Table S5. Unscaled wavenumbers λ in cm^{-1} and intensity *I* in $km.mol^{-1}$ obtained via harmonic frequency calculations for the *Z* conformer of F8-AZB at B3LYP-GD3BJ/6-31G(d,p) level in implicit solvation (ε=28.735).

λ	*I*	λ	*I*
17.4444	0.0173	671.8672	13.3682
38.1406	0.1634	694.1330	11.6609
39.4450	0.9024	712.0887	1.0484
90.2408	0.5626	718.9870	18.7793
109.6052	0.2613	724.7747	31.4884
131.9887	0.7022	824.1169	101.4891
134.1682	0.3587	840.0394	32.6040
148.8429	0.1980	848.5879	29.4680
208.8827	9.1028	963.4676	9.4464
222.8029	3.0809	976.7161	469.4485
242.1587	7.2982	1000.4956	89.8152
274.8175	0.4387	1017.8181	28.1451
275.3139	0.0861	1160.0611	0.8383
293.8094	1.6499	1161.4428	26.2719
294.7868	0.0115	1200.6518	15.7826
312.7694	3.5553	1201.8919	159.2507
314.6587	2.3191	1279.9999	184.5395
331.8196	7.1223	1284.4438	117.1903
357.8411	2.3643	1346.3790	32.9180
404.0887	6.8938	1355.6513	3.9461
411.3583	0.8886	1411.3410	100.2917
426.0919	0.9126	1415.9213	8.2331
430.5394	0.5406	1471.0363	37.2956
452.1932	3.7862	1472.3694	14.6156
453.4622	0.0035	1538.1001	1142.5047
490.1403	0.9519	1545.1233	151.6679
505.4332	2.1301	1608.1143	61.9616
551.0303	0.6363	1658.9380	71.5102
557.8953	0.0098	1665.5850	2.8326
585.3488	0.2634	1683.5336	8.8742
631.2158	4.5265	1684.6914	2.6047
638.1910	6.8266	3245.3786	16.7389
642.2725	0.9581	3245.4034	19.4067

Table S6. Unscaled wavenumbers λ in cm^{-1} and intensity I in $km.mol^{-1}$ obtained via harmonic frequency calculations for the *E* conformer of F8-AZB at B3LYP-GD3BJ/6-31G(d,p) level in implicit solvation (ε=28.735).

λ	I	λ	I
15.6519	0.0169	656.5371	0.0263
42.3190	0.9577	696.5896	1.5210
42.7949	2.0121	709.6996	19.5838
65.9760	0.4293	718.0530	0.1160
128.5557	0.0319	723.0775	10.7982
129.3769	0.0001	800.4851	2.9877
147.0806	0.1756	845.0778	1.6983
159.3492	4.2451	847.1442	83.7490
174.5162	1.4060	959.3625	5.3752
185.0460	2.4349	975.4809	598.3220
202.9124	0.2482	1083.7894	97.9916
248.2283	6.8082	1087.0171	2.7804
286.1783	0.1843	1158.2748	24.1729
286.7027	0.0067	1163.8583	0.1500
304.9204	0.0194	1202.0195	177.1105
306.5590	2.1876	1202.6039	3.1971
314.2923	0.0316	1293.9133	0.0073
348.6010	16.4589	1321.6224	205.9068
371.8232	0.0555	1345.6942	126.8994
377.9763	0.3353	1351.8902	1.6747
391.7263	7.0936	1403.2939	0.5148
407.5861	0.0038	1408.3689	532.2554
409.3799	0.4430	1461.4569	0.1324
432.3275	0.0004	1474.9530	0.6278
451.2399	8.6997	1522.5742	11.9981
472.9181	14.2499	1537.1839	1313.0799
501.5453	0.0000	1560.2490	9.5639
530.7620	1.3272	1653.3662	68.0388
570.5677	3.2614	1657.3140	0.7317
601.9964	68.4455	1680.9412	0.1055
642.6787	1.6367	1681.8405	6.1025
645.2882	0.0060	3242.9284	44.2162
649.0204	0.0524	3242.9611	0.0060

Table S7. Unscaled wavenumbers λ in cm^{-1} and intensity I in $km.mol^{-1}$ obtained via harmonic frequency calculations for the Z conformer of F10-AZB at B3LYP-GD3BJ/6-31G(d,p) level in implicit solvation (ε=28.735).

λ	I	λ	I
17.5654	0.0418	580.9809	0.0399
28.3599	0.0011	584.4073	0.2777
42.6386	0.4817	589.2670	0.0054
76.7018	2.0067	608.9941	2.5626
109.9337	0.3304	616.6061	11.3056
124.6451	0.3918	621.2988	2.1352
136.4254	0.4668	697.0915	7.5401
142.3045	0.0611	731.0968	19.1267
162.8704	0.5698	800.6750	1.7724
176.7926	0.0000	868.7513	36.4087
220.9415	12.1610	947.0105	125.5564
230.6499	4.6159	965.6013	153.8416
238.5442	9.6315	1016.9868	118.5942
261.6282	0.0487	1021.7120	632.0962
263.8988	1.0212	1137.3385	171.5344
266.0097	0.6556	1144.4725	128.3631
266.8131	0.4034	1174.4924	1.2483
299.5613	4.5140	1176.1255	1.1263
300.5941	0.2212	1331.3521	42.3713
316.5913	2.4071	1334.6342	38.0494
321.5161	4.2622	1339.9666	3.5029
350.9660	6.2183	1349.6697	0.3136
371.8647	0.1581	1434.5322	111.0617
379.0375	5.5155	1439.6790	1.3570
385.9262	1.4759	1536.6874	1092.9883
423.2551	5.7089	1543.6675	229.6347
442.5519	0.1399	1547.8118	235.0473
448.0572	0.6383	1548.4065	252.4002
454.3153	0.3474	1597.2119	115.6358
510.3196	1.6268	1665.6841	19.1394
533.0928	2.2624	1671.1707	0.2163
558.7744	0.1685	1687.5750	57.1782
579.5277	1.1899	1688.9749	45.8875

Table S8. Unscaled wavenumbers λ in cm^{-1} and intensity I in km.mol^{-1} obtained via harmonic frequency calculations for the *Z* conformer of F10-AZB at B3LYP-GD3BJ/6-31G(d,p) level in implicit solvation (ε=28.735).

λ	I	λ	I
20.0022	0.0059	573.3931	22.9592
35.5705	0.0000	583.9067	0.0020
41.4220	1.0014	610.3431	31.6632
64.2401	0.1711	625.4937	0.8853
131.2583	0.0874	625.5801	0.0002
132.4723	0.0007	631.9218	1.3599
140.6115	0.0916	685.0294	0.2891
151.8824	1.9375	709.9072	0.0011
154.8381	0.2363	806.2803	8.9062
173.4040	0.0059	834.9810	0.2638
180.3497	2.4676	977.0466	3.5144
193.3354	2.9865	1010.6911	298.5346
209.2063	0.1319	1021.0023	822.8155
261.9086	11.2601	1063.2288	6.2029
263.0515	0.3787	1160.0198	0.0827
264.8709	2.0794	1171.8760	3.2712
274.3895	0.4107	1187.3006	0.1231
274.9880	0.0752	1194.4633	142.2003
308.4315	0.0307	1332.5443	0.0537
310.1758	5.0611	1342.1435	77.7306
336.3481	6.7915	1349.8699	0.3975
348.4622	0.0000	1356.4580	116.5609
371.5601	0.0622	1421.4811	0.5697
378.9515	0.0017	1430.1298	688.2375
380.8240	1.4393	1518.7078	7.4511
407.5899	14.5848	1538.8884	1139.3424
442.7348	0.0012	1546.9565	2.4109
444.9220	56.4947	1548.9203	857.5798
451.3486	0.9240	1561.9138	5.1965
453.7074	0.5883	1660.2903	62.9809
495.7971	0.0076	1663.7002	0.0259
571.7243	19.4581	1684.4383	248.7524
573.0367	0.8674	1684.5102	0.1392

Determination of the F8-AZB$_x$@DMOF-1 and F10-AZB$_x$@DMOF-1 systems via liquid-state NMR and IR spectroscopy. For the determination of the composition of the F8-AZBx-@DMOF-1 and F10-AZBx@DMOF-1 systems, liquid-state NMR and IR spectroscopy were applied. In the following, a detailed description for the calculations is given, starting with F8-AZB as guest molecule.

F8-AZB$_x$@DMOF-1. Determination of the F8-AZB$_x$@DMOF-1 systems was performed via ^{1}H proton NMR taking the *E*/*Z* ratios obtained via IR spectroscopy into account. The ^{1}H NMR spectra are shown in Figures S13 to S15, the calculations considering the *E*/*Z* ratios obtained via IR spectroscopy are listed in Table S9.

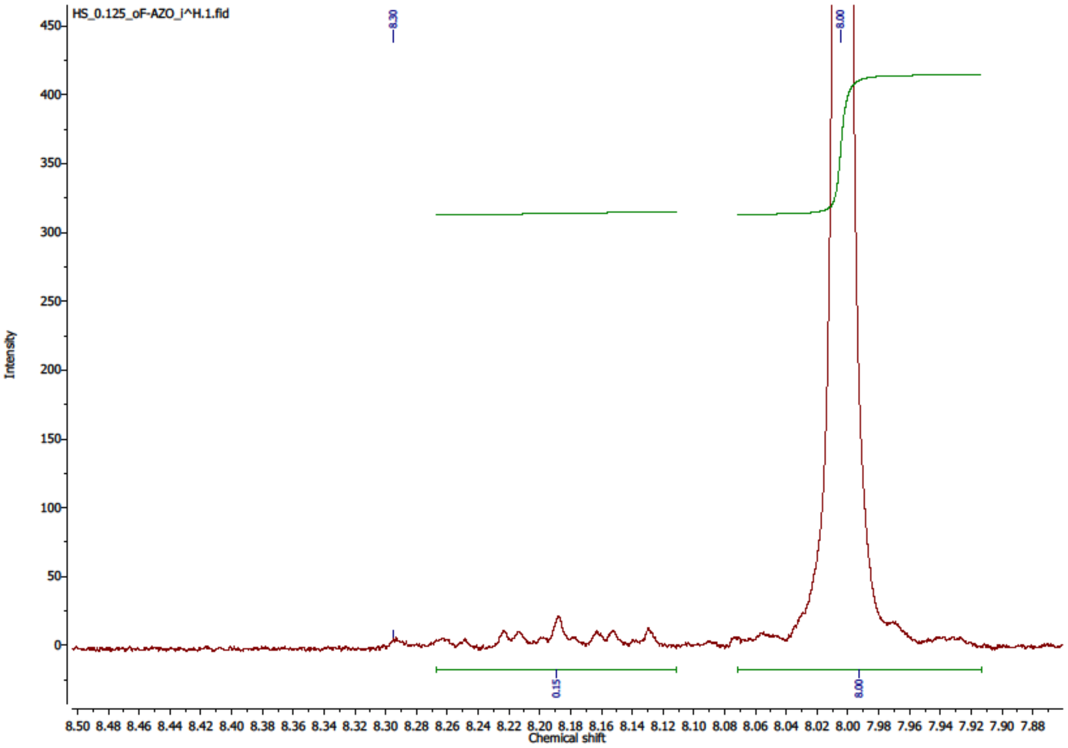

Figure S13. ^{1}H NMR spectrum of F8-AZB$_{0.125}$@DMOF-1 digested in 1 ml of DMSO-d_6 and 25 µl of DCl.

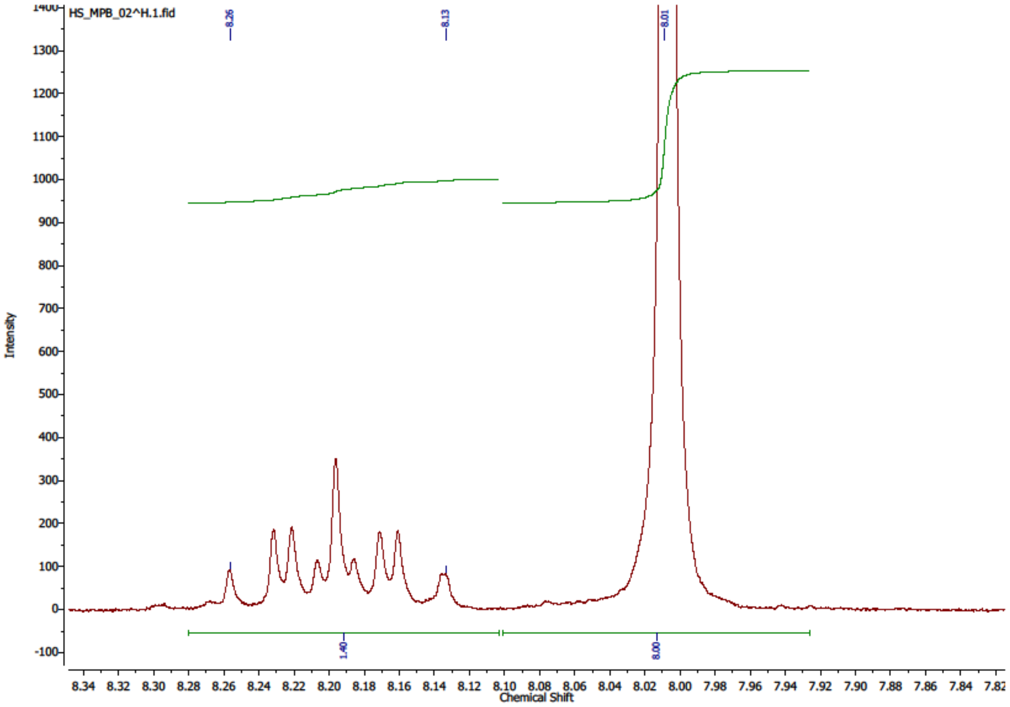

Figure S14. ^{1}H NMR spectrum of F8-AZB@DMOF-1 digested in 1 ml of DMSO-d_6 and 25 µl of DCl.

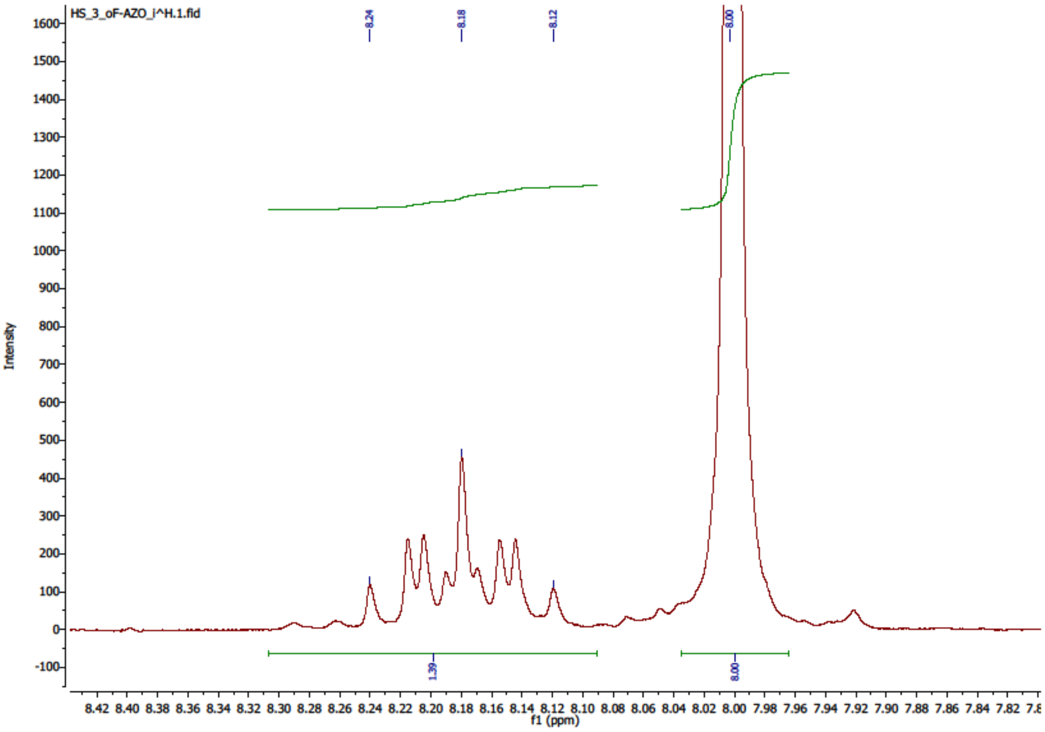

Figure S15. ^{1}H NMR spectrum of F8-AZB_3@DMOF-1 digested in 1 ml of DMSO-d_6 and 25 µl of DCl.

Table S9. Compositions of F8-AZB_x@DMOF-1 systems taking into account the isomer ratios in the initial state obtained via IR spectroscopy (Table 2, main article).

Quantity used	*E*/*Z* ratio in %	Peak area *E* protons	Peak area *Z* protons	x calculated
x = 0.125	59/41	0.15	0.104	0.125
x = 1	69/31	1.4	0.62	1.02
x = 3	56/44	1.39	1.09	1.24

For all F8-AZB_x@DMOF-1 systems, the obtained F8-AZB-to-MOF ratios are reasonable: Only for x = 3, resublimed F8-AZB during the synthesis procedure was observed. Therefore, the composition of this system was expected to be lower than the quantities used. With a value of x = 1.24, this assumption is confirmed.

Unfortunately, liquid-state NMR cannot be applied for the composition determination of the F10-AZBx@DMOF-1 systems. Further, as outlined in the main article, XPS is also not suitable, as the F10-AZB molecules start to sublime out of the pores under the required measurement conditions. Therefore, IR spectroscopy was applied. For this quantitative analysis, the peak intensities of the IR bands at

1057 cm^{-1} (peak 1) and 1016 cm^{-1} (peak 2) were compared. While the first band originates from the MOF host, the latter greatly overlaps with the band of F10-AZB. In Table S10, the calculations and corresponding results are listed.

Table S10. Compositions of F10-AZB_x@DMOF-1 systems taking into account the isomer ratios in the initial state obtained via IR spectroscopy (Table 2, main article).

Quantity used	Ratio peak heights peak 1 to peak 2 of DMOF-1	Ratio peak heights peak 1 to peak 2 of F10-AZB_x@DMOF-1	x calculated
x = 0.125	1:1.23	1:1.35	0.12
x = 1	1:1.23	1:3	**1.77**
x = 3	1:1.23	1:2.42	1.19

As visible for x = 1 as quantity used, the calculated value is too high, although the ratios used were exact as well as several measurements were performed. Here, a non-homogenous loading is assumed, which will be further investigated in on-going studies.

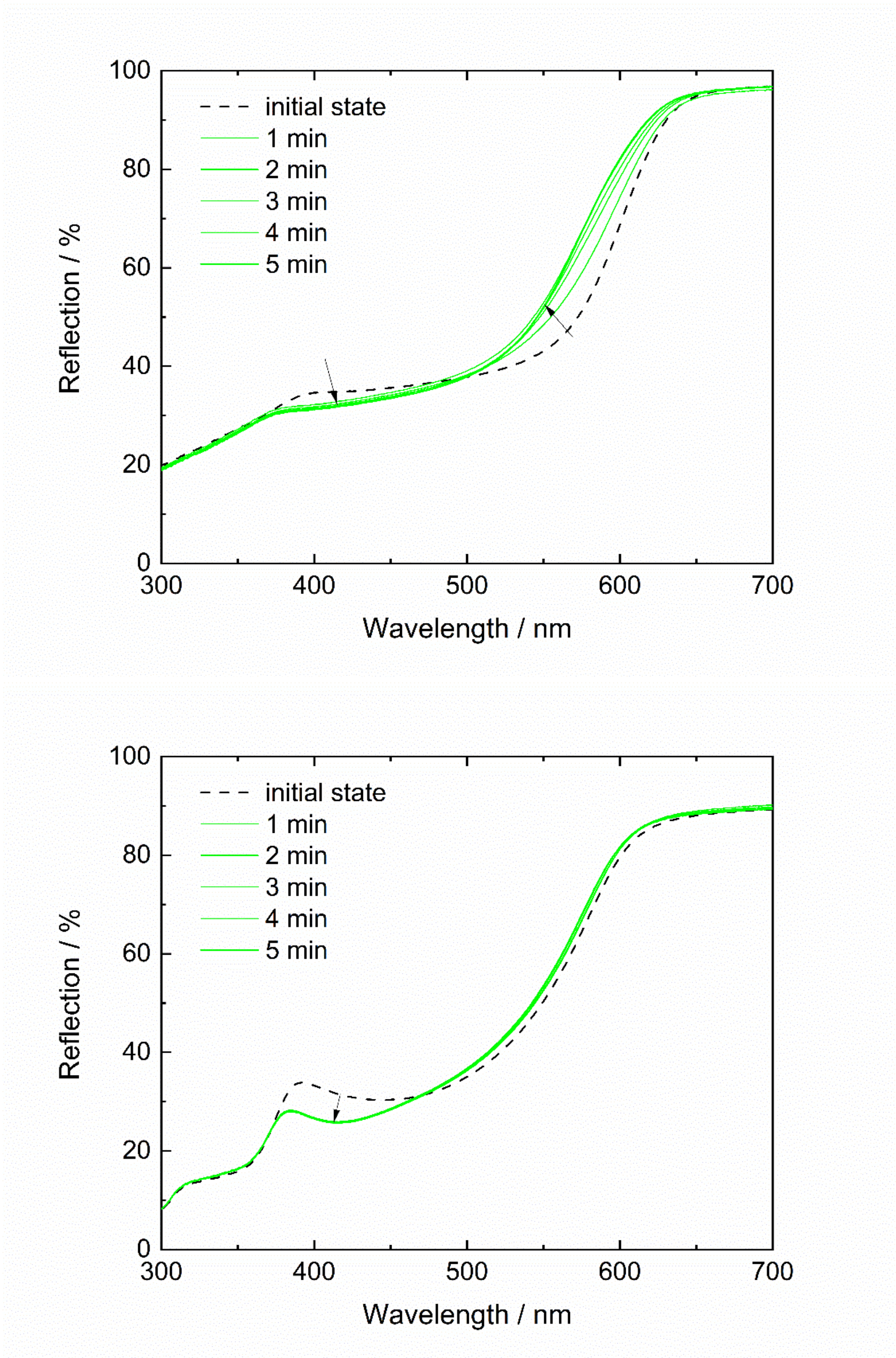

Figure S16. UV/Vis reflectance spectra of F8-AZB@DMOF-1 (top) and F10-AZB@DMOF-1 (bottom) before (dashed black lines) and after irradiation with and green light (green lines) for a total of 5 min.

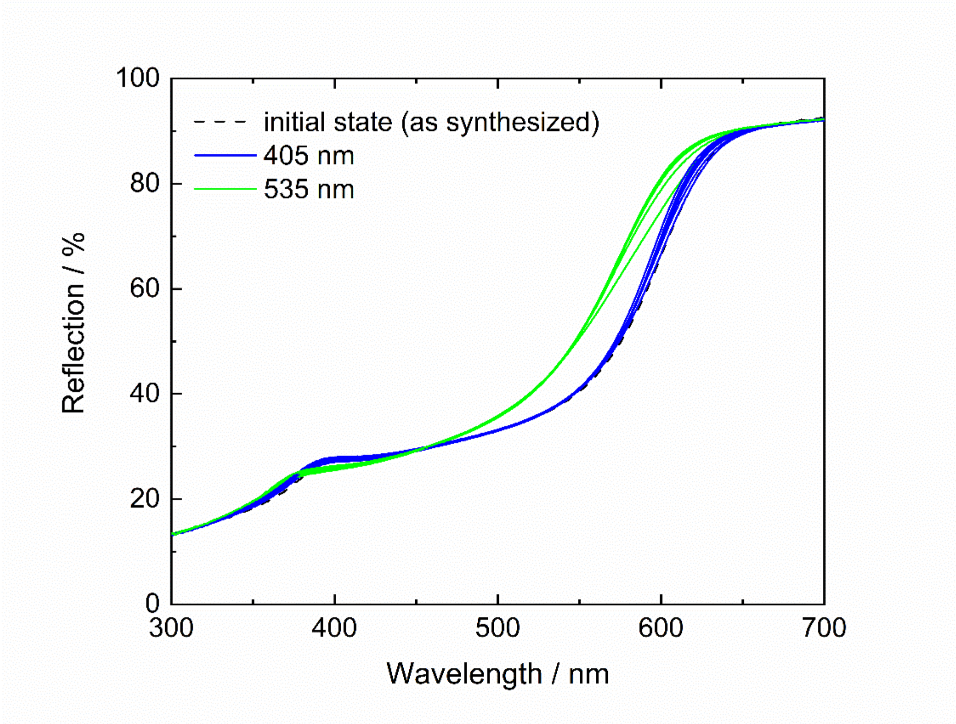

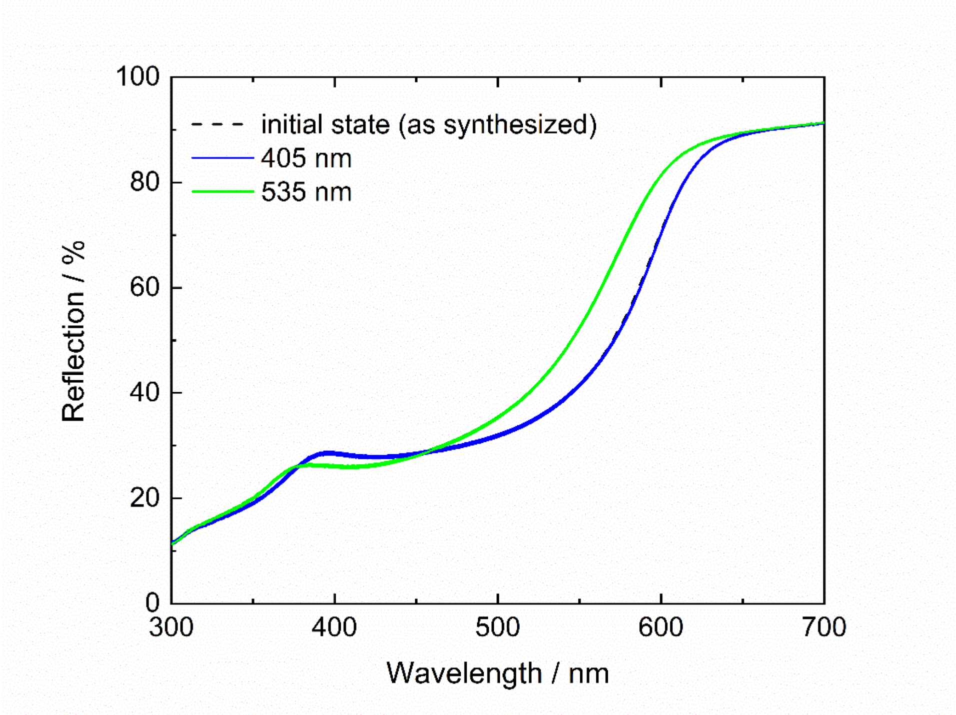

Figure S17. UV/Vis reflectance spectra of F8-AZB@DMOF-1 (top) and F8-AZB$_3$@DMOF-1 (bottom) before (dashed black lines) and after irradiation with violet (blue lines) and green light (green lines); ten switching cycles were performed.

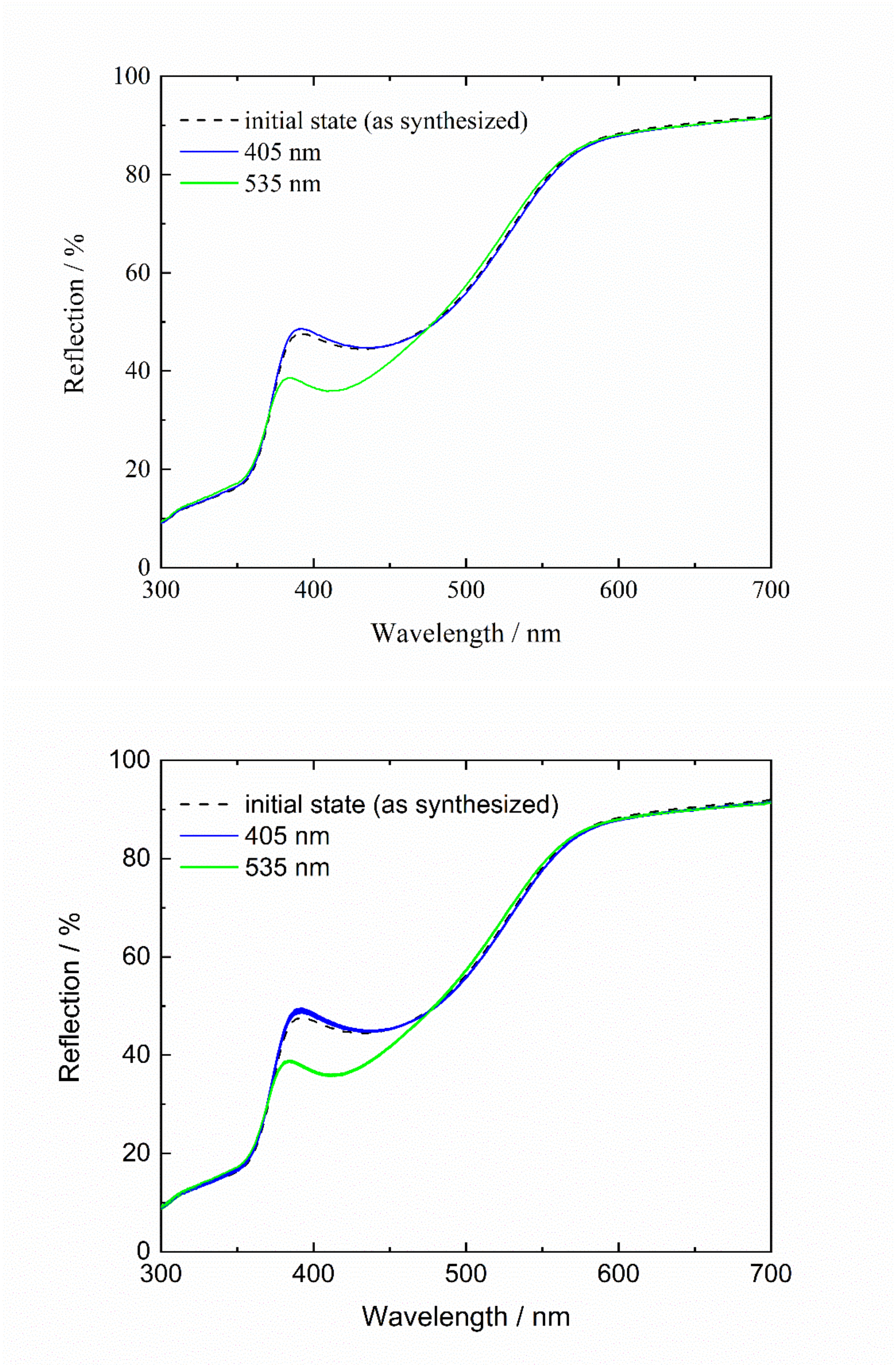

Figure S18. Top: UV/Vis reflectance spectra of F10-AZB$_{0.125}$@DMOF-1 before (dashed black line) and after irradiation with violet (blue line) and green light (green line); bottom: UV/Vis reflectance spectra of F10-AZB$_{0.125}$@DMOF-1 over ten switching cycles.

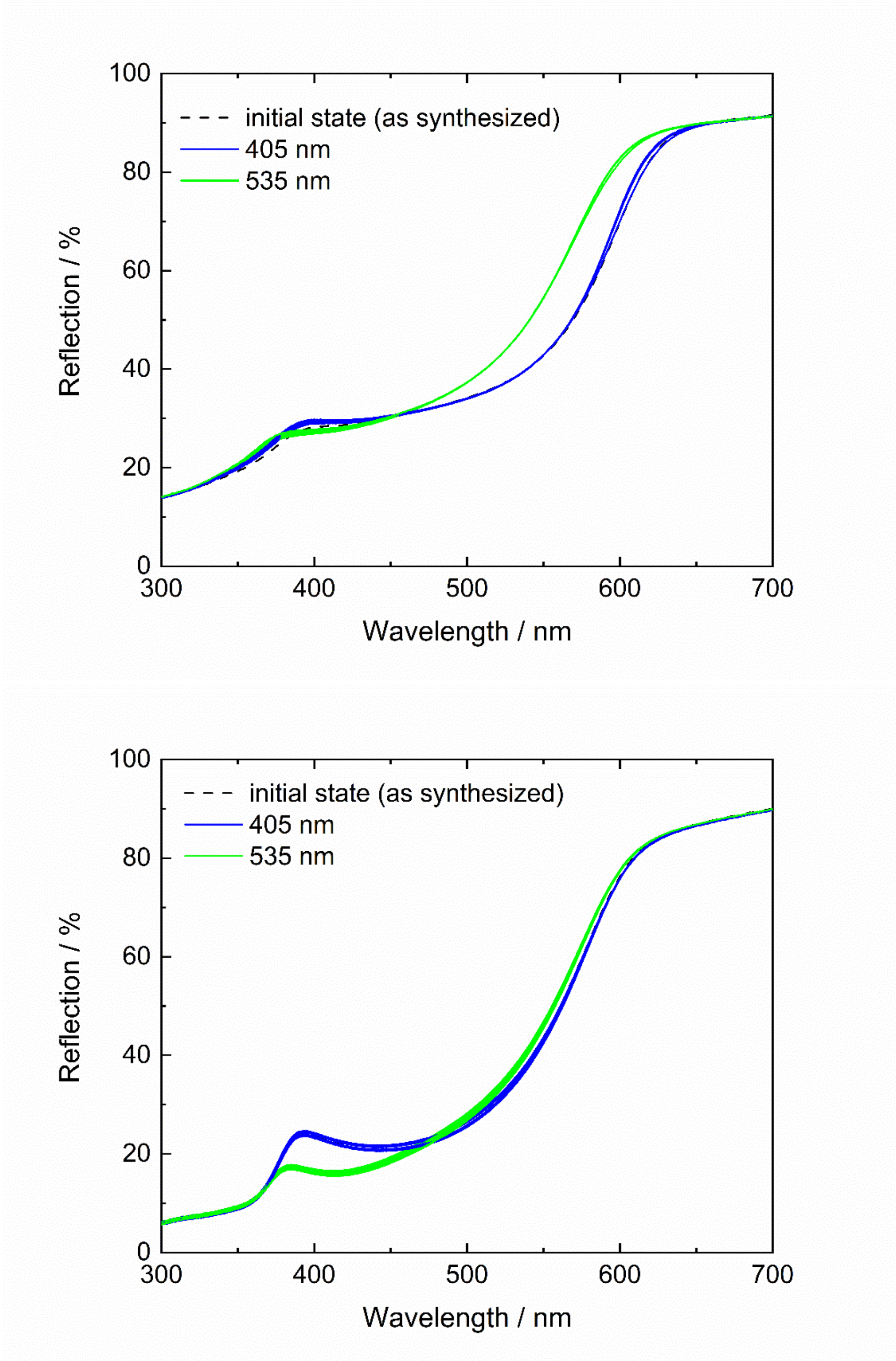

Figure S19. UV/Vis reflectance spectra of F10-AZB@DMOF-1 (top) and F10-AZB$_3$@DMOF-1 (bottom) before (dashed black lines) and after irradiation with violet (blue lines) and green light (green lines); ten switching cycles were performed.

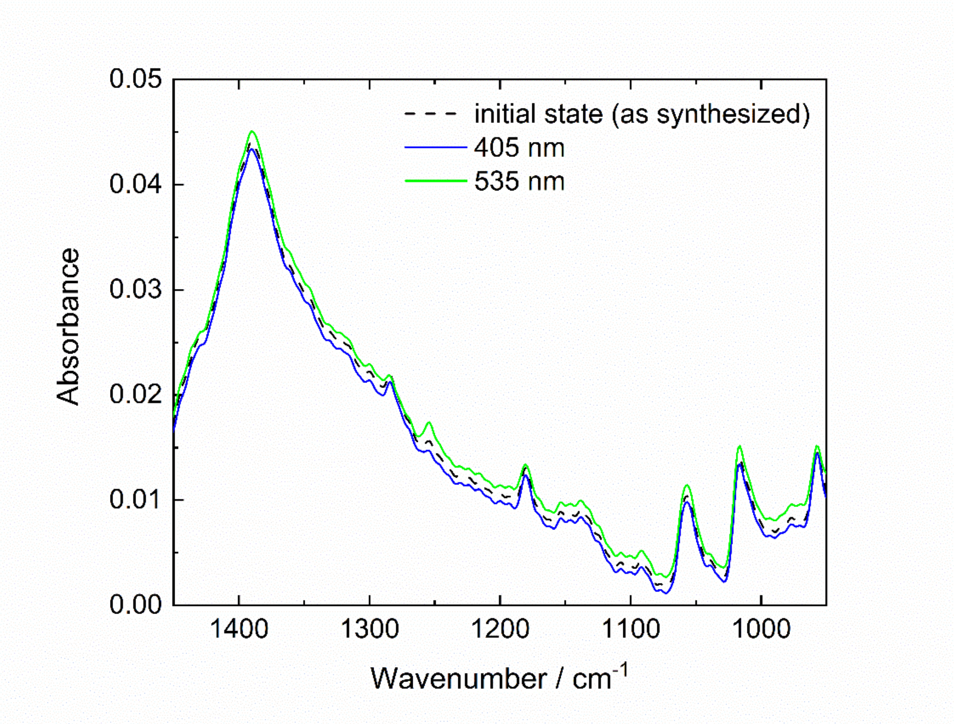

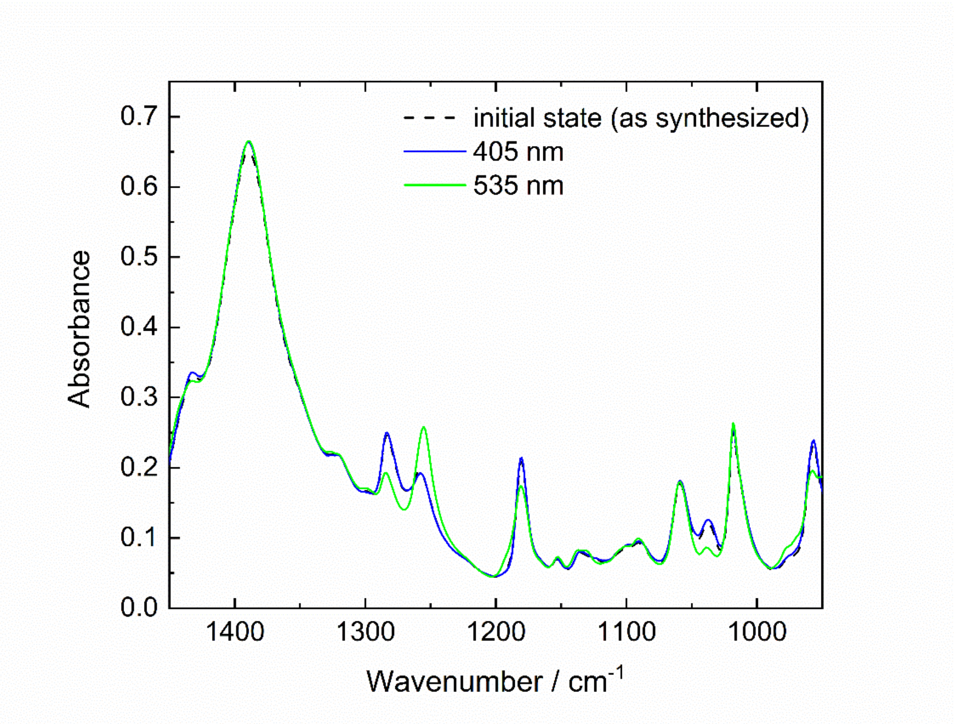

Figure S20. IR spectra of F8-AZB$_{0.125}$@DMOF-1 (top) and F8-AZB$_3$@DMOF-1 (bottom) before (dashed black lines) and after irradiation with violet (blue lines) and green light (green lines).

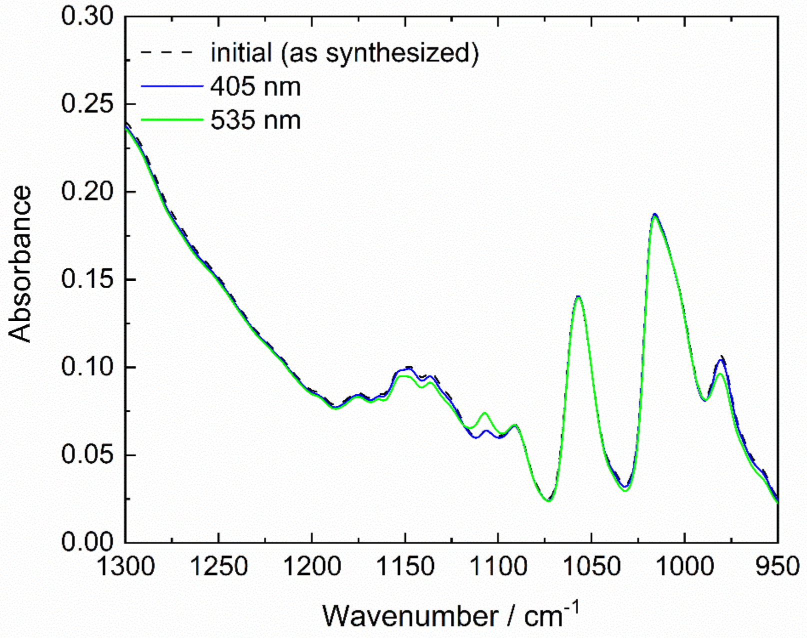

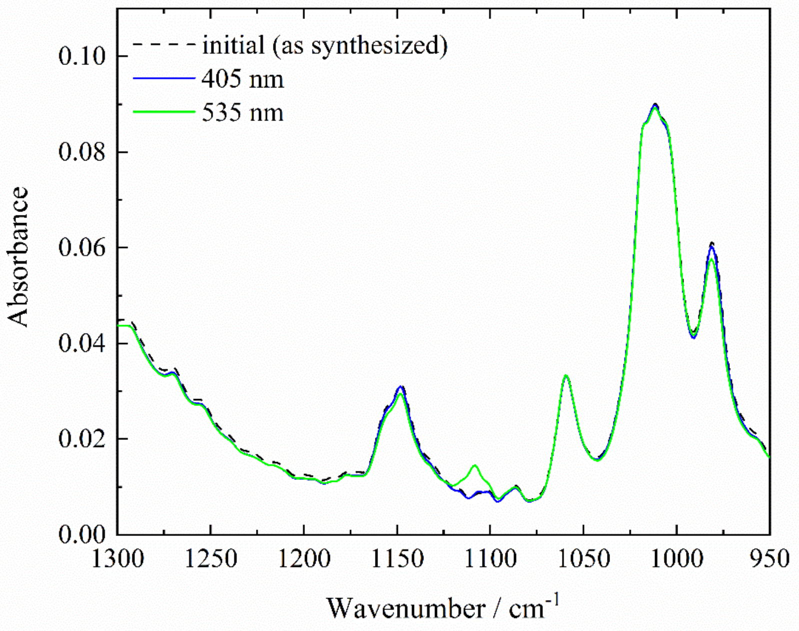

Figure S21. IR spectra of F10-AZB$_{0.125}$@DMOF-1 (top) and F10-AZB$_3$@DMOF-1 (bottom) before (dashed black lines) and after irradiation with violet (blue lines) and green light (green lines).

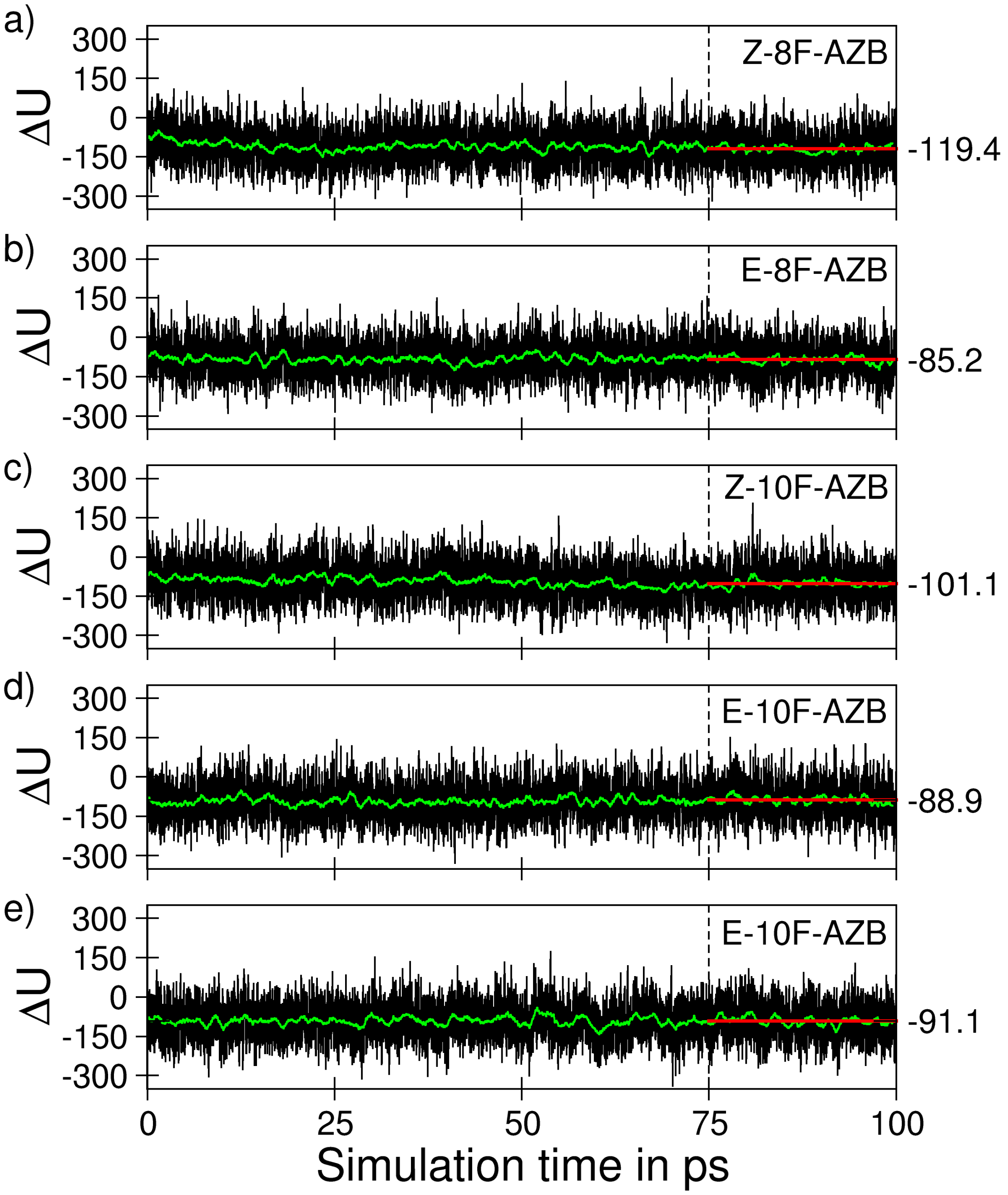

Figure S22. Time series of the instantaneous interaction potential ΔU_{int} (black) and the associated running average over 250 data points (green) obtained from the DFTB MD simulations of a) *Z*-F8-AZB, b) *E*-F8-AZB, and c) *Z*-F10-AZB as well as d) and e) two independent simulations performed for *E*-F10-AZB. The associated averages (red) have been evaluated employing only the last 25 ps of the simulation trajectories.

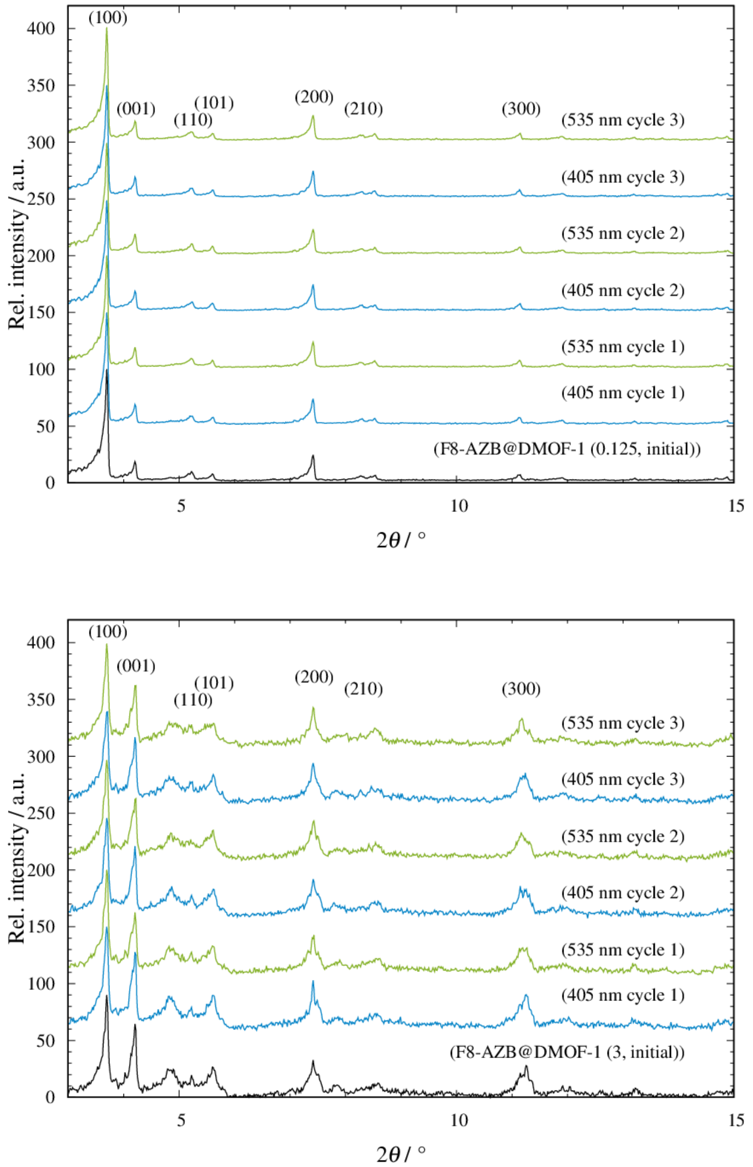

Figure S23. F8-$AZB_{0.125}$@DMOF-1 (top) and F8-AZB_3@DMOF-1 (bottom) before (black line) and after irradiation with violet (blue lines) and green light (green lines). Three switching cycles were performed. The diffraction patterns were measured at 298 K (*Stoe Stadi P*: $\lambda = 0.7093$ Å).

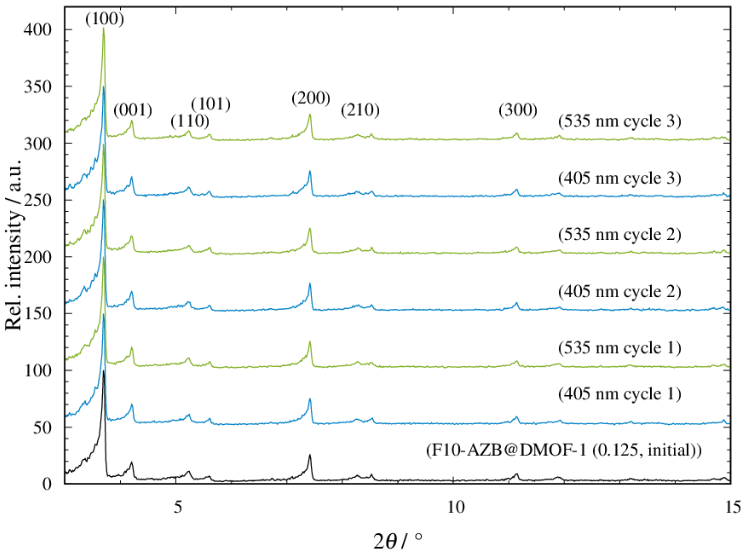

Rel. intensity / a.u.
2θ / °
(100)
(001)
(110)
(101)
(200)
(210)
(300)
(535 nm cycle 3)
(405 nm cycle 3)
(535 nm cycle 2)
(405 nm cycle 2)
(535 nm cycle 1)
(405 nm cycle 1)
(F10-AZB@DMOF-1 (0.125, initial))

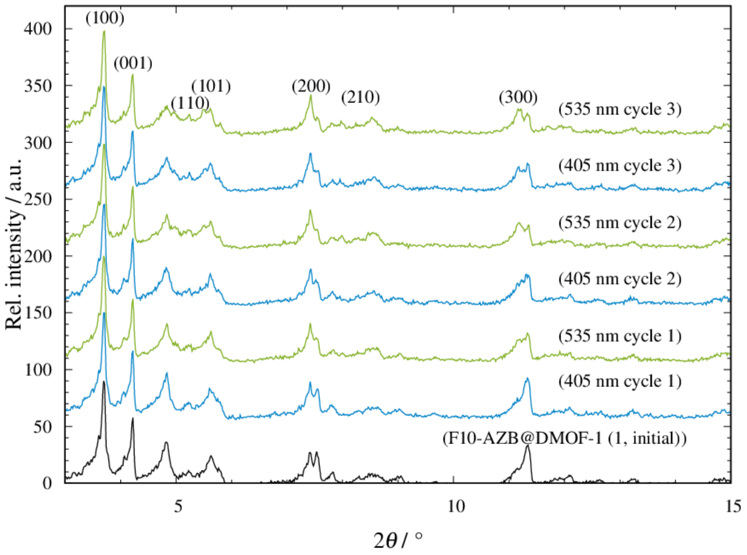

Rel. intensity / a.u.
2θ / °
(100)
(001)
(110)
(101)
(200)
(210)
(300)
(535 nm cycle 3)
(405 nm cycle 3)
(535 nm cycle 2)
(405 nm cycle 2)
(535 nm cycle 1)
(405 nm cycle 1)
(F10-AZB@DMOF-1 (1, initial))

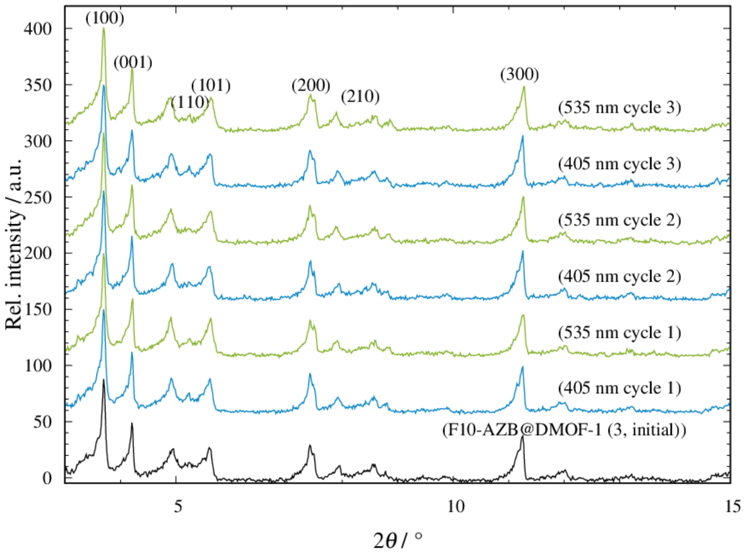

Rel. intensity / a.u.
2θ / °
(100)
(001)
(110)
(101)
(200)
(210)
(300)
(535 nm cycle 3)
(405 nm cycle 3)
(535 nm cycle 2)
(405 nm cycle 2)
(535 nm cycle 1)
(405 nm cycle 1)
(F10-AZB@DMOF-1 (3, initial))

Figure S24. F10-AZB$_{0.125}$@DMOF-1 (top), F10-AZB@DMOF-1 (center), and F10-AZB$_3$@DMOF-1 (bottom) before (black line) and after irradiation with violet (blue lines) and green light (green lines). Three switching cycles were performed. The diffraction patterns were measured at 298 K (*Stoe Stadi P*: λ = 0.7093 Å).
